# Inhibition of *C. albicans* Dimorphic Switch by Cobalt(II) Complexes with Ligands Derived from Pyrazoles and Dinitrobenzoate: Synthesis, Characterization and Biological Activity

**DOI:** 10.3390/ijms20133237

**Published:** 2019-07-01

**Authors:** Daniela Fonseca, Sandra M. Leal-Pinto, Martha V. Roa-Cordero, José D. Vargas, Erika M. Moreno-Moreno, Mario A. Macías, Leopoldo Suescun, Álvaro Muñoz-Castro, John J. Hurtado

**Affiliations:** 1 Department of Chemistry, Universidad de los Andes, Carrera 1 No. 18A-12, 111711 Bogotá, Colombia; 2 Grupo de Investigación en Manejo Clínico-CLINIUDES, Facultad de Ciencias de la Salud, Universidad de Santander, 680002 Bucaramanga, Colombia; 3 Grupo de Investigación en Biotecnología Agroambiente y Salud-MICROBIOTA, Facultad de Ciencias de la Salud, Universidad de Santander, 680002 Bucaramanga, Colombia; 4 Cryssmat-Lab, DETEMA, Facultad de Química, Universidad de la República, Av. 18 de Julio 1824-1850, 11200 Montevideo, Uruguay; 5 Grupo de Química Inorgánica y Materiales Moleculares, Universidad Autónoma de Chile, El Llano Subercaseaux, Santiago 2801, Chile

**Keywords:** pyrazole and dinitrobenzoate ligands, cobalt(II) complexes, crystal structure, *Trypanosoma cruzi*, antifungal activity, cytotoxicity, dimorphic switch

## Abstract

Seven cobalt(II) complexes of pyrazole derivatives and dinitrobenzoate ligands were synthesized and characterized. The single-crystal X-ray diffraction structure was determined for one of the ligands and one of the complexes. The analysis and spectral data showed that all the cobalt complexes had octahedral geometries, which was supported by DFT calculations. The complexes and their free ligands were evaluated against fungal strains of *Candida* *albicans* and emerging non-*albicans* species and epimastigotes of *Trypanosoma cruzi*. We obtained antifungal activity with a minimum inhibitory concentration (MIC) ranging from 31.3 to 250 µg mL^−1^. The complexes were more active against *C. krusei,* showing MIC values between 31.25 and 62.5 µg mL^−1^. In addition, some ligands (**L1**–**L6**) and complexes (**5** and Co(OAc)_2_ · 4H_2_O) significantly reduced the yeast to hypha transition of *C. albicans* at 500 µg mL^−1^ (inhibition ranging from 30 to 54%). Finally, the complexes and ligands did not present trypanocidal activity and were not toxic to Vero cells. Our results suggest that complexes of cobalt(II) with ligands derived from pyrazoles and dinitrobenzoate may be an attractive alternative for the treatment of diseases caused by fungi, especially because they target one of the most important virulence factors of *C. albicans.*

## 1. Introduction

Microorganisms, such as fungi and parasites, cause diseases that have become a public health problem worldwide. Fungal infections caused by opportunistic yeasts of the genus *Candida* are becoming more frequent and are associated with high morbidity and mortality [1]. *C. albicans* is the most frequently isolated species in patients with candidiasis; however, the emergence of non-albicans strains, such as *C. tropicalis, C. parasilopsis, C. krusei,* and *C. glabrata*, among others, has become a serious problem due to the varied sensitivity of these strains to antifungals and to a lack of timely diagnosis [2,3]. Azoles, amphotericin B and echinocandins have been used as common antifungals for many years, but adverse effects, prolonged therapies and resistance, among others, limit treatment options [1].

On the other hand, the *T. cruzi* parasite causes Chagas disease, a zoonotic parasitosis that is predominantly vectorially transmitted through a hematophagous insect [4]. Treatment of this disease is limited because since the 70s only two drugs, Nifurtimox and Benznidazole, have been used, both of which cause serious side effects. In the context of the compounds used for the treatment of these diseases, azoles stand out [5]. Azoles inhibit the enzyme lanosterol 14 α-demethylase, which participates in the biosynthesis of ergosterol. In the same way, benzoate ligands play an important role in bioinorganic chemistry because the carboxylate group is very versatile, and they have high antifungal activity. The study of azole chemistry represents a promising option, and the development of ligands derived from azoles bound to transition metals has been of particular interest due to the increased biological activity of the complexes relative to that of the free azoles [6,7]. Our research group previously studied the antibacterial and antifungal activities of cobalt(II), chromium(III) and copper(II) complexes derived from benzotriazole and triazole, which showed higher antimicrobial activities than the free ligands [8]. Likewise, studies of the trypanocidal activity of cobalt(II), copper(II), zinc(II), nickel(II) and chromium(III) complexes with pyrazole derivatives were also carried out, which showed higher activity with respect to the corresponding ligands. Similarly, Batista et al. reported that manganese(II), cobalt(II) and copper(II)-1,10-phenanthroline complexes derived from norfloxacin (NOR) and sparfloxacin (SPAR) had higher trypanocidal activity than free NOR and SPAR drugs [9]. The increase in the biological activity of the complexes may be due to polarity reduction, which is generated due to the metal–ligand union, to the overlap of the orbitals of the ligand and the metal and the contribution of electrons of the donor groups on the deficient metal. That results in an increase in its lipophilic character, favoring permeation through the lipid membrane and affecting certain normal processes inside the cell. In this work, we report the synthesis and characterization of new cobalt(II) complexes derived from pyrazole ligands and 3,5-dinitrobenzoic acid and their antifungal, trypanocidal and cytotoxic activity [10,11].

## 2. Results and Discussion

**Figure 1:**
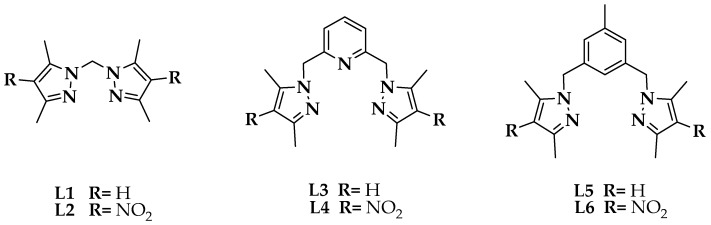
Azole ligands under study.

A systematic study of the structure and biological activities of the azole ligands (**L1**–**L6**) in Figure 1 was synthesized as described in the literature, and their Co(II) complexes prepared under different conditions were performed. The synthesis of azole **L6** was not previously reported so we synthesized and characterized it.

### 2.1. Synthesis and Characterization of 2,6 bis(4-nitro-3,5-dimethylpyrazol-1-ylmethyl)pyridine **L6**

The synthesis of **L6** was performed by a modified literature procedure [12]. **L6** was synthesized by the reaction between 4-nitro-3,5-dimethylpyrazole and 3,5-bis(bromomethyl)toluene in toluene. **L6** was isolated as an air-stable white solid with an 80% yield by crystallization from dichloromethane: ethyl ether. The ligand was characterized by elemental analysis, Raman, infrared, ultraviolet/visible, RMN spectroscopy and mass spectrometry (Figures S1–S35 in the Supplementary Materials). The FT-IR spectra showed bands for asymmetric and symmetric stretching of the NO_2_ group at 1485 and 1350 cm^−1^, respectively. The methylene group was observed at δ 53.31 ppm (Figure S7 in the Supplementary Materials). The ^13^C and ^1^H chemical shifts were assigned with the aid of a heteronuclear single quantum correlation (HSQC) experiment. The ^1^H NMR spectra showed six singlets, and the signal corresponding to the proton in the pyrazole ring was not observed, indicating that substitution of H for NO_2_ had occurred (Figure S6 in the Supplementary Materials). Additionally, the signals were observed at a lower field than the corresponding singlets in **L5** [13], which is expected because the NO_2_ group is an electron withdrawing substituent. The crystal structure of **L5** is discussed later in this manuscript. 

### 2.2. Synthesis and Characterization of the Cobalt(II) Complexes 

**Figure 2:**
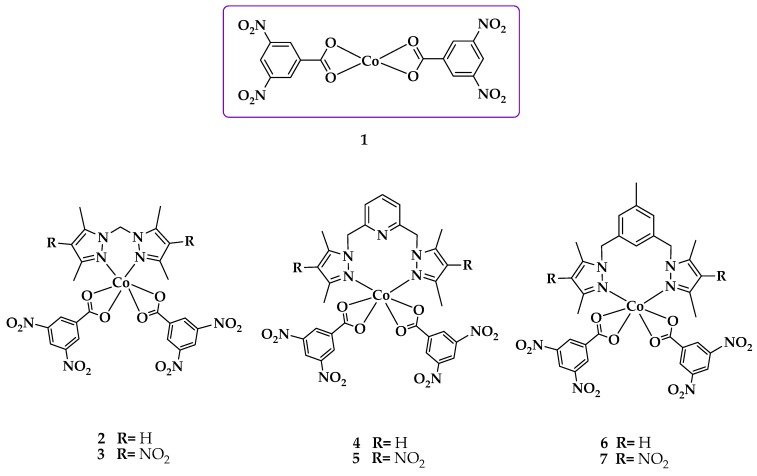
Possible structure of the complexes under study.

The precursor complex [Co(dnb)_2_] (**1**) was synthesized by the reaction between 3,5-dinitrobenzoic acid and Co(OAc)_2_ ∙ 4H_2_O in MeOH at a 2:1 molar ratio, respectively. In the case of complexes **2**–**7**, the respective ligand (**L1**–**L6**) was mixed with **1** using two solvents in which both were dissolved. However, **2** was the only complex synthesized using only one solvent in the reaction. The cobalt complexes (Figure 2) were stable in air at room temperature and were isolated in high yields. Table 1 shows the elemental analysis, melting point, and color of complexes. 

The results allowed us to propose a 1:2 [Metal(M):Co-ligand(dnb)] relative stoichiometry for **1**. Complexes **1**–**6** showed a 1:1:2 [M:azole ligand(**L**):(dnb)] ratio, resulting in a [M(**L**)(dnb)_2_] type stoichiometry.

#### 2.2.1. FT-IR and Raman Spectroscopy

The complexes were analyzed by infrared spectroscopy to observe the shifts of defined bands relative to those of the free ligands following coordination to the metal center. The bands corresponding to the vibrations of the most representative bonds of **L1**–**L6** and the precursor complex [Co(dnb)_2_] (**1**) were compared with those observed in the FT-IR spectra of their respective complexes **2**–**7**. Table 2 summarizes the assignments of the most significant vibration bands.

Ligands **L1**, **L3** and **L5** are ligands that do not have the nitro group in their structure, whereas **L2**, **L4** and **L6** do have it (Figure 1). When comparing the FT-IR spectra of **2**–**7** with those of **L1**–L**6** (Figures S1–S5, S11 and Table S1 in the Supplementary Materials), it was observed that the band associated with the vibration of the asymmetric stretch υ(C–H) of the methyl groups, was present in all azole ligands and complexes in the same position. However, the band corresponding to the vibration of the stretch υ(C=C) of the pyrazole ring was observed as shifted to a lower wave number in all complexes compared to free ligands [14]. This is because the bonds are less rigid; therefore, they require less energy to vibrate. The band associated with the vibration of the stretch of the bond υ(C=N) of the pyrazole ring appears in **2**–**7** displaced towards greater number of waves, which implies that the bonds vibrate to greater energy; this means that they have a greater rigidity in the complexes. This result suggests that the ligands are being coordinated to the metallic center by the N-pyrazole. Another band identified in the complexes was associated with the bending vibration in the pyrazole ring plane in the range of 802 to 825 cm^−1^ [14].

**Figure 3:**
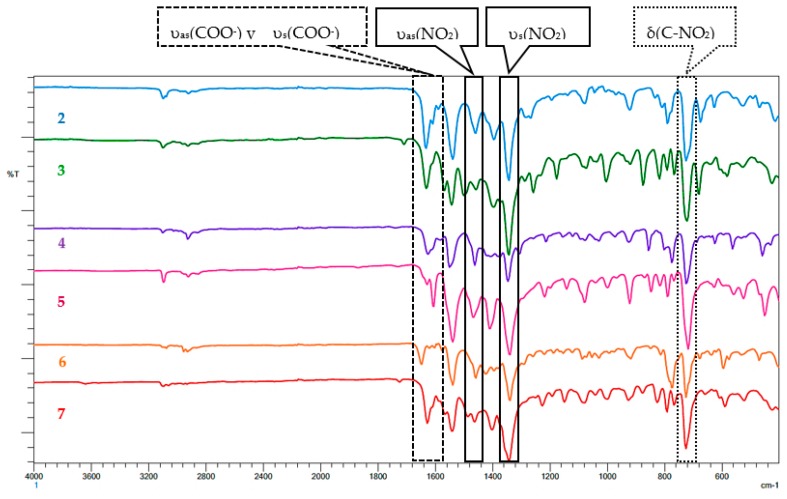
Comparison of absorption spectra in the infrared region of complexes **2**–**7**.

The asymmetric stretching υ_as_(NO_2_) between 1458 and 1500 cm^−1^ and symmetric stretching υ_s_(NO_2_) between 1338 and 1346 cm ^−1^ corroborate the presence of NO_2_ in all the complexes and suggest that NO_2_ is not directly involved in coordination [12]. In the case of **3** and **4**, the bands corresponding to the υ(C-H) vibration of the pyridine ring at 1045 (**3**) and 1041 (**4**) cm^−1^ do not show shifts relative to the free ligands **L3** and **L4**, which indicates that the nitrogen of the pyridinic ring is not coordinated to the metallic center [15]. In addition, in the FT-IR spectra of **1**–**7**, two bands associated with asymmetric and symmetric stretching of the carboxylate group were observed. To determine the coordination form of the carboxylate in the complexes (monodentate, bridged and/or bidentate), Δυ = υ_as_(COO^−^) − υ_s_(COO^−^) values were calculated. In all the complexes, these Δυ values were very low, which indicates bidentate chelation of carboxylates [16]. The mentioned bands for the carboxylate group and nitro group are indicated in Figure 3. However, the band associated with the vibration υ_s_ (COO^−^) was not explicitly indicated in Figure 3, due to its low intensity. However, it appears in the range of 1604 to 1566 cm^−1^ in all complexes.

**Figure 4:**
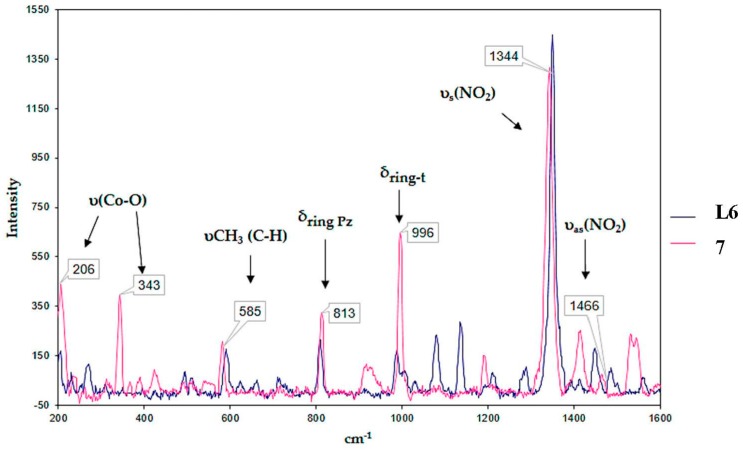
Comparison of absorption spectra in the Raman region of **L6** and **7**.

In the Raman spectra of the complexes, **2**–**7** can be bands that come from the azole ligand, in addition to bands corresponding to the coordination of this ligand to the metallic center. As an example, Figure 4 shows the comparison of the Raman spectrum of **7** with ligand **L6**. Two bands corresponding to the azole ligands were identified. The band of the stretching vibration υ(C–H) of the methyl groups between 527 and 600 cm^−1^ and the band corresponding to the bending vibration in the pyrazole ring plane in the range of 811 to 820 cm^−1^. Additionally, bands related to **1** were observed. Two bands corresponding to the υ_as_(NO_2_) asymmetric stretching between 1400 and 1462 cm^−1^ and υ_s_(NO_2_) symmetric stretching between 1344 and 1365 cm^−1^ were present. In addition, the band corresponding to the 1,3,5-trisubstituted benzene ring was observed between 993 and 996 cm^−1^. Finally, the band assigned to the stretching vibration of the υ(Co–O) bond was observed in a range of 201 to 348 cm^−1^. The presence of these bands confirms coordination of the benzoate ligands with the metallic center, corroborating the formation of **2**–**7**, as described above.

#### 2.2.2. UV/Vis Spectroscopy

The UV-Vis spectra of **1** was recorded in MeOH, and two bands are observed in the ultraviolet region, which can be attributed to intraligand transitions: the first band at 205 nm corresponding to transitions between the *n*-π* orbitals, and the second at 233 nm corresponding to transition of the π-π* orbitals of the dinitrobenzoate ligand. However, the transfer bands are not evident due to an overlap with the absorption bands characteristic of the ligand dnb. In addition, the UV-Vis spectra of **2**–**6** were recorded in DMSO (dimethyl sulfoxide), and a band between 200 and 350 nm in the ultraviolet region was observed corresponding to transitions between the π–π*orbitals of the ligand. Charge transfer bands were also observed in this zone. Charge transfer transitions of the metal to the ligand mostly occur because the complexes contain ligands with π* orbitals of low energy, which can accept electron density from the metal. An important feature found was the absence of *n*–π * bands in all the complexes due to the formation of the N–M bond that stabilizes the pair of electrons in the nitrogen atoms. Additionally, all complexes **1**–**7** showed other bands corresponding to ***d****–**d*** transitions in a range of 400 to 700 nm. These bands correspond to the *^4^T_1g_ (F)* *→ ^4^A_2g_ (F)* electronic transition characteristic of *d^7^* metal complexes.

The UV-Vis spectra of **2**–**6** are not directly comparable to the spectrum of **1** because they were recorded in different solvents. In the spectra of **2**–**6,** a bathochromic shift was observed relative to the spectrum of **1**. In comparing the spectra of complexes **2**, **4** and **6** to those of **3**, **5** and **7**, respectively, a bathochromic shift was observed, likely due to insertion of the nitro group that extends the conjugation of the aromatic ring and generates a shift to lower energy due to the unsubstituted ring.

To verify the stability of complexes (**1**–**3**) in DMSO solution, the electronic (UV-Vis) spectra of these complexes were recorded as a function of time. No significant variations were observed, suggesting that the formed species of complexes in DMSO solution are stable in the assayed conditions [17].

#### 2.2.3. X-Ray Structural Determination of **L5** and **1**

**Figure 5:**
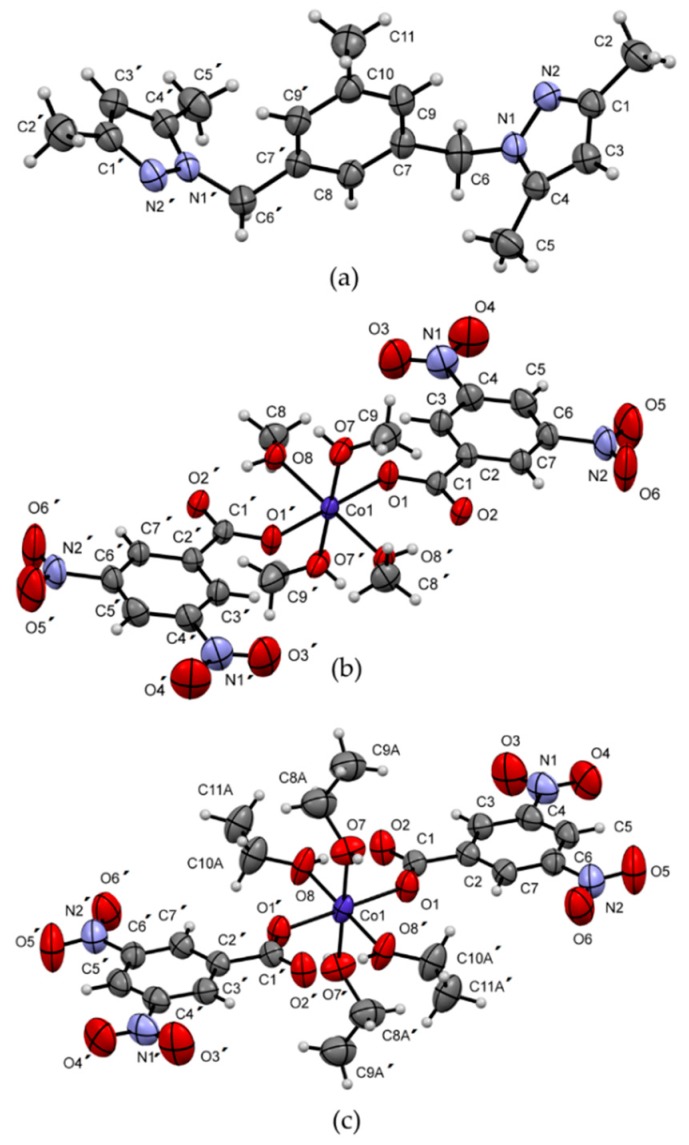
Molecular structures of (**a**) **L5** and complex **1** recrystallized in (**b**) methanol and (**c**) acetone: ethanol, showing anisotropic displacement ellipsoids drawn at the 50% probability level. H atoms are shown as small spheres of arbitrary radii. Selected bond lengths (Å) for (**b**): Co(1)‒O(1) 2.0671(14), Co(1)‒O(7) 2.0710(15), Co(1)‒O(8) 2.1119(13) and (**c**): Co(1)‒O(1) 2.0612(18), Co(1)‒O(7) 2.098(2), Co(1)‒O(8) 2.086(2). Selected bond angles (°) for (**b**): O(1)‒Co(1)‒O(7) 88.77(6), O(1)‒Co(1)‒O(8) 90.75(5) and for (**c**): O(1)‒Co(1)‒O(7) 90.40(8), O(1)‒Co(1)‒O(8) 89.75(8).

Figure 5 shows the molecules of ligand **L5** and complex **1** coordinated by methanol and ethanol, and Table 3 shows the crystal data and experimental details. The molecular structure of **L5** shows two methyl substituted pyrazole rings joined through a ‒CH_2_‒ group to a central toluene fragment. The structure, which crystallizes in the Monoclinic *C*2/*c* space group, has a Z’ value of 0.5 due to a coincident symmetry of the molecule with the two-fold rotation axis along the [010] direction that falls in the center of the molecule at (0, y, 1/4). This axis makes the two pyrazole fragments symmetrically equal and oriented in a *cis*‒conformation, forming a dihedral angle of 88.61(6)° with the central six-membered ring, which is different from the *trans*-conformation observed in the organic-inorganic hybrid (LH)^+^(FeCl4)^−^, where L = 3,5-bis(3,5-dimethylpyrazole-1-ylmethyl)toluene [18]. In the supramolecular structure, no classical hydrogen bonds were found. Instead, a combination of weak C11‒H11B···N2 hydrogen interactions (distance C11···N2 of 3.865(2) Å) connecting molecules along the [−101] direction and van der Waals forces are responsible for the observed three-dimensional network.

The crystal structure of complex **1** was also analyzed by X-ray crystallography. Interestingly, in our attempts to obtain crystals of suitable quality, two different crystal structures were found. Recrystallization in methanol (**1*m***) and acetone: ethanol (**1*e***) gave the crystal structures shown in Figure 5b,c, with methanol and ethanol coordinating the central Co atom, respectively. In both structures, a mononuclear six-coordinate complex was observed. The coordination sphere contains six oxygen atoms from four alcohol molecules and two dinitrobenzoate ligands in an octahedral geometry with average volumes of 12.044 Å^3^ and 12.018 Å^3^, bond angle variances of 2.05° and 1.71° for **1*m*** and **1*e****,* respectively, and identical mean quadratic elongations of λ = 1.001 [19]. Complexes 1***m*** and 1***e*** crystallize in the triclinic *P*−1 and monoclinic *P*2_1_/*n* space groups, respectively, with Z´ values of 0.5 due to a coincident symmetry of the molecules with an inversion center. The dinitrobenzoate groups act as monodentate ligands with the O2 atoms connected by intramolecular O8‒H8···O2 (H···O distances of 1.89(2) Å and 1.85(3) Å for **1*m*** and **1*e****,* respectively) hydrogen bonds to the coordinating alcohol molecules. This form of coordination results differently from that observed in the FT-IR analysis considering that, in this case, the crystallographic study was performed on a recrystallized sample. The supramolecular structure is dominated by strong pairs of equivalent O7‒H7···O2 hydrogen bonds (H···O distances of 1.90(3) Å/1.90(2) Å and symmetry codes 1 − x, 1 − y and −z/1 + x, y, z for **1*m*** and **1*e***, respectively) connecting pairs of inversion-related molecules to form chains running along the [100] direction in both structures. The three-dimensional networks are completed through van der Waals forces. Interestingly, in both crystal structures, the molecules have similar arrangements and intermolecular interactions. During refinements, half of the ethanol molecules showed positional disorder, which presumably breaks the 2-fold symmetry axes and the glide planes observed in the monoclinic structure (methanol), making the unit cell of **1*m*** nearly double the volume of that of **1*e***. 

**Figure 6:**
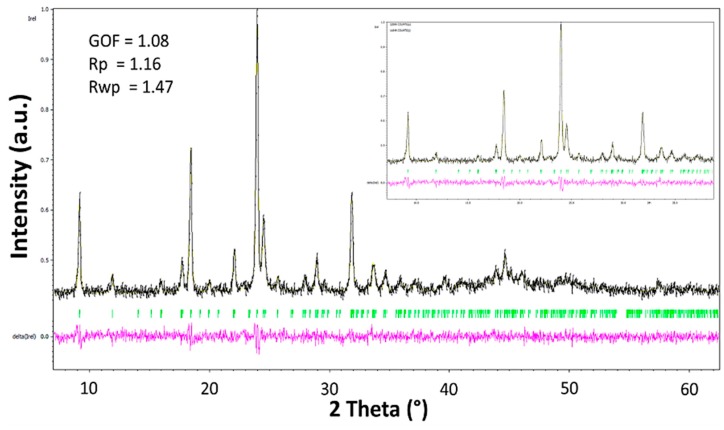
Graphical result of LeBail analysis after heat treatment of complex **1** at 130 °C.

Thermal treatment of complex **1** (pink) at 130 °C for 8 h produced a purple powder. Several attempts to grow single crystals from this powder sample were unsuccessful. Regardless of the solvent used, the metallic center always adopted an octahedral geometry involving solvent molecules. In this sense, the square planar geometry of the cobalt complex in the purple sample was lost during recrystallization. Therefore, X-ray powder diffraction analysis was carried out. Efforts to resolve the structure from powder data were fruitless. However, autoindexing of the diffraction peaks and unit cell refinement by Le Bail analysis [20] showed that the whole diffractogram for this complex is explained by the unit cell *a* = 9.6586 (17) Å, *b* = 11.6717 (14) Å, *c* = 6.3204 (9) Å and β = 96.286 °, with the most likely space group *P*2_1_/*m* (Figure 6) in a monoclinic symmetry. It is important to observe that the purple powder corresponds to a pure sample since extra peaks from the secondary phases were not observed.

### 2.3. Quantum-Chemical Calculations

To explore relevant physicochemical properties along the series and to rationalize differences in their biological activity, density functional theory (DFT) calculations were carried out at the dispersion corrected TZ2P/BP86-D3 level of theory [21] with all-electron basis sets via the ADF code [22]. The obtained relaxed structures exhibited an octahedral coordination sphere for the studied systems, where the calculated structure for **1** agrees with characterization via X-ray measurements. In this concern, the permanent molecular dipole moment (µ) has appeared to be a useful parameter in drug–receptor interaction within a quantitative structure–activity relationship (QSAR) framework since early works [23] since it plays a crucial role in promoting long-range electrostatic interactions for supramolecular structure stabilization and drug-site interactions in biomolecules [19].

**Figure 7:**
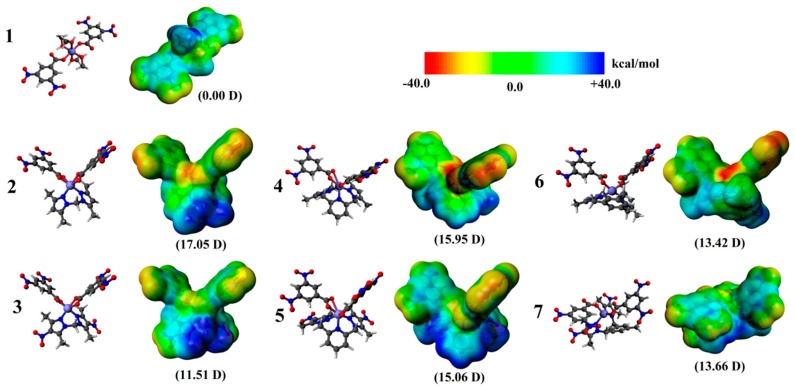
Relaxed structures, molecular electrostatic potential (MEP) energy surface and calculated dipole moment on a surface set at 0.001 a.u.

Our results denote differences in the calculated molecular dipole moment, increasing from **1**, with a zero dipole moment owing to its centrosymmetric structure, to values larger than 11 Debyes, which is accounted by the charge distribution on an electron density surface of 0.001 a.u. (electrons/Bohr^3^) showing a van der Waals surface of each molecule [24,25] as given by the molecular electrostatic potential (MEP) energy surface (Figure 7). Previously, we qualitatively correlated the lowest MIC values with larger permanent dipole moments (µ values) in the series [8] when the isolated ligands did not exhibit relevant biological activity. In this series, all of the Co(II) species showed mild biological activity against C. *albicans*, C. *tropicalis* and C. *krusei* (Table 4). Introduction of nitro-groups into isolated ligands and Co(II) complexes enables rather similar biological activity owing to their inherent enzymatic reduction function [26]. Thus, a similar mechanism involving the enzymatic reduction role of –NO_2_ groups is expected.

### 2.4. Biological Activity

The discovery of new compounds with an anti-*Candida* effect is pivotal to combat candidiasis, a major opportunistic fungal infection with increasing morbidity and mortality worldwide [1]. Although *C. albicans* is the most frequent etiology, there has been an alarming global emergence of non-albicans strains due to their resistance to the common antifungals used [3]. First, we tested cytotoxic activity using an MTT assay, which determines mitochondrial function in cells by measuring mitochondrial enzymes, indicators of cell viability [27]. The compounds in this study were not toxic to Vero cells. We obtained CC_50_ values ranging from 103.97 to >300 µg mL^−1^, except **L5** (CC_50_ = 44.95 µg mL^−1^), which showed cytotoxic activity (Table 4). Itraconazole used as reference drug showed toxicity higher than 93% of the compounds assayed.

Then, the biological activity was confirmed with fungal and parasite cells to examine therapeutic potential, considering that some cobalt complexes have been studied as antimicrobial agents and have shown potential activity against different strains of fungi and *T. cruzi* [5,12,28,29]. The complexes analyzed exhibited antifungal activity against at least two of the three *Candida* species utilized in this study. We obtained MIC values ranging from 31.3 to 250 µg mL^−1^. By contrast, the free ligands were not active against these strains (MIC > 2000 µg mL^−1^) (Table 4). The increase in the biological activity of the complexes may be due to increases in their lipophilicity, causing polarity reduction of the fungal membrane and enhancing antimicrobial activity [8,30,31].

The compounds were more active against *C. krusei*. In this case, **3** showed fungicidal activity at 31.25 µg mL^−1^ and the best selective index (SI = 9.55), followed by **6** and **7** (SI = 4.79 and 4.13, respectively). Although multidrug resistance is uncommon in non-albican*s Candida* species, recently, it has been reported with more frequency [1,2,3]. In particular, *C. krusei* is known to be intrinsically resistant to Fluconazole, the main drug used to treat Candida infection [32], which probably increases their prevalence as etiological agents of invasive candidiasis and candidemia, as well as non-albicans isolates less susceptible to commonly used antifungal agents. Thus, the anti-Candida profile of the complexes synthesized shows promise for further development of preclinical assays, and it is necessary to elucidate their effect on virulence and pathogenicity factors. 

**Figure 8:**
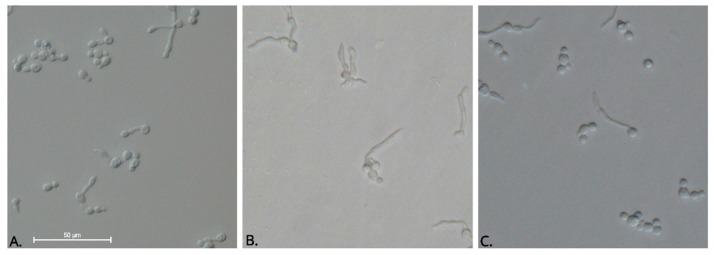
Effect of **L1** on *C. albicans* germ tube formation**.** Pooled human serum samples were inoculated with *C. albicans* yeast and incubated for 3 h at 37 °C. Morphology was assessed and photographed with a Nikon Eclipse Ni phase-contrast microscope (40×)**. A**. Itraconazole; **B**. Negative control; **C**. **L1** at 500 µg mL^−1^.

On the other hand, we tested the inhibitory activity of the complexes and their ligands against dimorphic switch of *C. albicans*. The morphological switch to hyphae is critical to pathogenesis, especially because the hyphal form has been shown to be more invasive than the yeast form due to the expression of cell-wall proteins that facilitate adhesion and tissue invasion [33,34]. Interestingly, we found that 83.3% of free ligands and 25% of the complexes synthesized inhibited tube germ formation (Table 5). Complexes **4** and **7** showed less than 20% inhibitory activity at the maximum concentration assayed. The remaining complexes (50%) were not active against the yeast to hypha transition (data not shown). Itraconazole was tested as the reference drug. In our hands, the average percent of filamentation inhibition that it presented was 44% at 1.0 µg mL^−1^. However, there were no significant differences between the range of concentrations evaluated in the positive control (*p* = 1.00). One-way ANOVA followed by Tukey’s post hoc test established that the effect of **L1** was better than the inhibition produced by Itraconazole at 0.5 and 0.25 µg mL^−1^ (*p* < 0.01) and equivalent to that produced by Itraconazole at 1 µg mL^−1^ (*p* = 0.051) (Figure 8). Although antifungal susceptibility experiments suggested that the free ligands did not inhibit yeast cell growth, they inhibited filamentous growth, which could provide evidence concerning the therapeutic potential of these compounds because strains unable to perform the phase transition are less virulent [35,36]. The signal transduction pathways and key transcription factors that modulate the dimorphic transition in *C. albicans* have been previously established [37,38,39,40,41,42,43]. In this study, we used human serum as an inducer of the morphological change from yeast to hyphae, and thus the inhibitory activity observed might suggest that cobalt(II) complexes with ligands derived from pyrazoles and dinitrobenzoate impact the activity of the Ras-mediated signal transduction pathway, which is involved in serum-induced filamentous growth [44]. However, to investigate the effect of the free ligands and complexes on hyphae-inducing signaling pathways, further experiments are required. 

In addition, we tested the effect of the compounds against *T. cruzi* epimastigotes. Although previous studies conducted by our group with similar molecules (metal complex derivatives of bis (pyrazol-1-yl) methane ligands) suggested antiparasitic potential of these compounds [12], in this study, we did not observe trypanocidal activity at the maximum concentration assayed (IC_50_ > 100 µg mL^−1^). Benznidazole showed an IC_50_ value of 18.4 µg mL^−1^ + 1.3.

## 3. Materials and Methods

### 3.1. General Information

The starting compounds 3,5-dinitrobenzoic acid (ADNB), cobalt(II) acetate tetrahydrate Co(OAc)_2_ ∙ 4H_2_O, 3,5-bis(bromomethyl)toluene (BBMT), and tetrabutylammonium bromide (TBAB) were used as received from Alfa Aesar (Ward Hill, MA, USA). The solvents that were required to be anhydrous were dried, distilled and stored on 3 Å molecular sieves under a nitrogen atmosphere before use. Elemental analyses (C, H and N) were performed with a Thermo Scientific^™^ FLASH 2000 CHNS/O Analyzer (Thermo Fisher Scientific, Waltham, MA, USA). Fourier transform infrared (FT-IR) spectra were recorded on a Shimadzu IR Tracer-100 spectrophotometer (Shimadzu Corporation, Kyoto, Japan) in a range of 400–4000 cm^−1^ using KBr pellets. Melting points were recorded with a capillary Mel-Temp^®^ 1101D Electrothermal apparatus in open capillary tubes and are uncorrected (Staffordshire, UK). Nuclear magnetic resonance (NMR) spectra were recorded on a Bruker Ascend^™^-400 spectrometer (Bruker, Billerica, MA, USA) at 295 K (400.13 MHz for ^1^H; 100.61 MHz for ^13^C) in the solvent CDCl_3_. ^1^H and ^13^C NMR chemical shifts (δ) are reported in parts per million (ppm) with the residual solvent peak used as an internal reference. High-resolution mass spectra (HRMS) were recorded on an Agilent Technologies 1260 (Q-TOF 6520) spectrometer (Agilent Technologies, Santa Clara, CA, USA) via electrospray ionization (ESI) in positive ion mode. The mass spectra were recorded on a Thermo Scientific™ TRACE™ 1300 Gas Chromatograph via electronic impact (Waltham, MA, USA). The electronic UV/ Vis absorption spectra were measured from 200 to 800 nm in DMSO solution in a quartz cuvette with a 1-cm optical path length using a Varian Cary 100 spectrophotometer from Agilent Technologies (Agilent Technologies, Santa Clara, CA, USA). Raman spectroscopy was performed in a HORIBA Scientific spectrometer (HORIBA Scientific, Kyoto, Japan) using a 785-nm laser in a range of 200–1600 cm^−1^. Recrystallization of **L5** was carried out by slow evaporation from a toluene solution. Complex **1** was recrystallized from solutions of methanol and a mixture of acetone: ethanol (1:1). These procedures afforded crystals of suitable size and quality for single-crystal X-ray diffraction. The data collection carried out using a Bruker D8 Venture/Photon 100 CMOS diffractometer (Madison, WI, USA) and refinement details are summarized in Table 3. In the refinements, all the nonhydrogen atoms were anisotropically treated, and the hydrogen atoms were generated geometrically, placed at geometrically suitable calculated positions (C–H = 0.93–0.97 Å) and refined by applying isotropic displacement parameters set at 1.2–1.5 times the U_eq_ value of the parent atom. The H atoms belonging to methanol and ethanol molecules in the coordination sphere of the Co atom in **1** were located in the difference Fourier maps and freely refined. The crystal structures were refined using the SHELXL2014 program [45]. The graphic material was prepared using Mercury 3.10.3 software [46].

A powder sample of complex **1**, thermally treated, was analyzed using X-ray powder diffraction at room temperature with an Empyrean-Panalytical X-ray diffractometer (Malvern Panalytical, Almelo, Netherlands) working in Bragg–Brentano geometry with Cu-Kα1,2 (1.5406 and 1.54439 Å) wavelengths. The diffractometer was operated over an angular range of 2θ = 2°–70° with a step size of 0.02° (2θ). Data analysis was performed by the Le Bail method using the Jana-2006 program [47]. The process of refinement was carried out assuming a pseudo-Voigt function for peak shape and a calculated background using a linear interpolation between a set of fixed points.

The ligands bis(3,5-dimethyl-1-pyrazolyl)methane (**L1**) [48], bis(3,5-dimethyl-4-nitro-1-pyrazolyl)methane (**L2**), 2,6-bis(3,5-dimethyl-1-pyrazolyl)pyridine (**L3**), 2,6-bis(3,5-dimethyl-4-nitro-1-pyrazolyl)pyridine (**L4**), and 3,5-bis(3,5-dimethylpyrazol-1-ylmethyl)toluene (**L5**) [12,48,49] were synthesized as described in the literature.

### 3.2. Synthesis of 3,5-bis(3,5-dimethyl-4-nitropyrazol-1-ylmethyl)toluene **L6**

This ligand was prepared by a modified literature procedure [12]; 4-nitro-3,5-dimethylpyrazole (1.8 mmol, 0.254 g), KOH (3.517 mmol, 208.43 g) and water (1 mL) were stirred at room temperature (rt) for 30 min, and then, tetrabutylammonium bromide (TBAB) (0.055 mmol; 0.018 g), 3,5-bis(bromomethyl)toluene (0.9 mmol, 0.250 g) and 30 mL of toluene were added. The mixture was heated to reflux for 26 h. The resulting mixture was evaporated to dryness, and the solid residue was extracted with CH_2_Cl_2_: water. The organic layer was collected and dried with anhydride sodium sulfate and evaporated to dryness. The white solid was crystallized from dichloromethane (DCM): ethyl ether. Yield: 0.286 g (80%). M.p.: 187–186 °C. IR (KBr, **υ**/ cm^−1^): 2924, 1562, 1485, 1400, 1350, 999. Raman (**υ**/ cm^−1^): 1448, 1351, 1137, 1079, 990, 808, 589. ^1^H NMR (400 MHz, CDCl_3_): δ 6.86 (s, 2H), 6.70 (s, 1H), 5.18 (s), 4H), 2.54 (s, 6H), 2.53 (s, 6H), 2.31 (s, 3H). ^13^C NMR (100 MHz, CDCl_3_): δ 146.3, 140.3, 140.0, 135.9, 131.5, 127.4, 122.5, 53.3, 21.4, 14.1, 11.7. DEPT-135: 127.47, 122.50, 53.31, 21.42, 14.19, 11.74. Anal. calcd. for C_19_H_22_N_6_O_4_: C, 57.28; H, 5.57; N, 21.09%; found: C, 57.28; H, 5.56; N, 21.07%. HRMS (ESI+) *m/z*: calcd. for [C_19_H_22_N_6_O_4_ + H] ^+^ 399.1780; found: 399.1836 [M + H] ^+^. UV-Vis (DMSO) [(concentration, M) λ_máx_, nm (Log ε, M^−1^ cm^−1^)]: (9.89 × 10^−5^) 260 (4.28). MS (EI) *m/z* calcd. for [C_14_H_16_N_3_O_2_]: 258.1; found [C_14_H_16_N_3_O_2_]: 258.1.

### 3.3. Synthesis of the Cobalt(II) Complexes (**1**–**7**)

#### 3.3.1. Synthesis of bis(dinitrobenzoate-O,O′) of Co(II) ([Co(dnb)_2_]) (**1**)

A solution of cobalt(II) acetate tetrahydrate Co(OAc)_2_∙4H_2_O (1 mmol, 0.25 g) in methanol (MeOH) (10 mL) was added to a solution of 3,5-dinitrobenzoic acid (DNBA) (2 mmol, 0.43 g) in MeOH (10 mL). The mixture was stirred at rt for 30 min. The solvent was evaporated to dryness to give a pink solid, which was recrystallized from MeOH. The solid obtained was dried at 130 °C for 8 h. Yield: 0.458 g (95%). M.p.: >400 °C. IR (KBr, **υ**/cm^−1^): 3097, 1624 1346, 725. Raman (**υ**/cm^−1^): 1539, 1350, 1190, 999, 918, 817, 346, 206. Anal. calcd. for C_14_H_6_CoN_4_O_12_: C, 34.95; H, 1.26; N, 11.64%; found: C, 34.90; H, 1.25; N, 11.51%. HRMS (ESI+) *m/z*: calculated for [C_14_H_8_N_4_O_12_Co + NH_4_] ^+^ 500.9814; found: 500.9129 [M + NH_4_] ^+^. UV-Vis (MeOH) [(concentration, M) λ_máx_, nm (Log ε, M^−1^ cm^−1^)]: (3.25 × 10^−4^) 205 (3.58), 233 (3.45); (2 × 10^−4^) 518 (1.01).

#### 3.3.2. Synthesis of Dinitrobenzoate [bis(3,5-dimethylpyrazol-1-yl)methane] of Co(II) ([Co(**L1**)(dnb)_2_]) (**2**)

A solution of **1** (0.42 mmol, 0.20 g) in MeOH (10 mL) was added to a solution of **L1** (0.40 mmol, 0.08 g) in toluene (10 mL). The mixture was stirred at rt for 12 h. The solvent was evaporated to dryness to give a pink solid, which was washed with ethanol (EtOH) and ethyl ether and dried at 100 °C for 8 h. Yield: 0.251 g (92%). IR (KBr, **υ**/cm^−1^): 3097, 2920, 1631, 1539, 1396, 1342, 1006, 725. Raman (**υ**/cm^−1^): 1537, 1348, 1187, 1050, 996, 915, 811, 588, 339, 201. Anal. calcd. for C_25_H_22_CoN_8_O_12_: C, 43.81; H, 3.24; N, 16.35%; Found: C, 43.80; H, 3.22; N, 16.33%. UV-Vis (DMSO) [(concentration, M) λ_máx_, nm (Log ε, M^−1^ cm^−1^)]: (3.32 × 10^−4^) 245 (3.42); (9.95 × 10^−4^) 543 (1.36). 

#### 3.3.3. Synthesis of Dinitrobenzoate[bis(3,5-dimethyl-4-nitro-pyrazol-1-yl)methane] of Co(II) ([Co(**L2**)(dnb)_2_]) (**3**)

A solution of **1** (0.42 mmol, 0.20 g) in acetone (10 mL) was added to a solution of **L2** (0.40 mmol; 0.1 g) in acetone (10 mL). The mixture was heated to reflux for 8 h. The solvent was evaporated to dryness to give a pink solid, which was washed with acetone: pentane (20:10) and then with EtOH and ethyl ether and dried at 100 °C for 8 h. Yield: 0.123 g (90%). IR (KBr, **υ**/cm^−1^): 3079, 2924, 1631, 1543, 1500, 1373, 1342, 1002, 721. Raman (**υ**/cm^−1^): 1540, 1465, 1351, 1189, 998, 813, 600, 341, 208. Anal. calcd. for C_25_H_20_CoN_10_O_16_: C, 38.72; H, 2.60; N, 18.06%; Found: C, 38.64; H, 2.60; N, 17.99%. UV-Vis in DMSO [(concentration, M) λ_máx_, nm (Log ε, M^−1^ cm^−1^)]: (3.45 × 10^−4^) 265 (3.28); (9.77 × 10^−4^) 552 (1.70).

#### 3.3.4. Synthesis of Dinitrobenzoate[2,6-bis(3,5-dimethylpyrazol-1-ylmethyl)pyridine] of Co(II) ([Co(**L3**)(dnb)_2_]) (**4**)

A solution of **1** (0.36 mmol, 0.17 g) in ethyl acetate (10 mL) was added to a solution of **L3** (0.34 mmol, 0.1 g) in DCM (10 mL). The mixture was heated to reflux for 7 h. The solvent was evaporated to dryness to give a pink solid, which was washed with CHCl_3_: n-heptane (50:50) and then ethyl acetate: n-heptane (40:10) and finally with ethyl ether and dried at 100 °C for 8 h. Yield: 0.208 g (80%). IR (KBr, **υ**/cm^−1^): 3093, 2962, 2873, 1643, 1608, 1573, 1462, 1427, 1396, 1369, 1342, 999, 725. Raman (**υ**/cm^−1^): 1535, 1460, 1344, 1044, 996, 816, 758, 582, 335, 212. Anal. calcd. for C_31_H_27_CoN_9_O_12_: C, 47.95; H, 3.50; N, 16.23%; found: C, 47.83; H, 3.48; N, 16.18%. UV-Vis (DMSO) [(concentration, M) λ_máx_, nm (Log ε, M^−1^ cm^−1^)]: (3.50 × 10^−4^) 267 (3.38); (9.88 × 10^−4^) 541 (1.12).

#### 3.3.5. Synthesis of Dinitrobenzoate[2,6-bis(3,5-dimethyl-4-nitro-pyrazol-1-ylmethyl)pyridine] of Co(II) ([Co(**L4**)(dnb)_2_]) (**5**)

A solution of **1** (0.28 mmol, 0.13 g) in ethyl acetate (10 mL) was added to a solution of **L4** (0.26 mmol, 0.1 g) in toluene (10 mL). The mixture was heated to reflux for 7 h. The solvent was evaporated to dryness to give a pink solid, which was washed with CHCl_3_: n-heptane (50:50), ethyl acetate: cyclohexene (20:30) and then with ethyl ether and dried at 100 °C for 8 h. Yield: 0.183 g (82%). IR (KBr, **υ**/cm^−1^): 3093, 2931, 1627, 1566, 1543, 1485, 1462, 1400, 1342, 999, 725. Raman (**υ**/cm^−1^): 1535, 1460, 1344, 1044, 996, 816, 758, 582, 335, 212. Anal. calcd. for C_31_H_27_CoN_11_O_16_: C, 42.97; H, 2.91; N, 17.78%; found: C, 42.69; H, 2.90; N, 17.73%. UV-Vis (DMSO) [(concentration, M) λ_máx_, nm (Log ε, M^−1^ cm^−1^)]: (3.42 × 10^−4^) 268 (3.50); (9.80 × 10^−4^) 544 (1.07).

#### 3.3.6. Synthesis of Dinitrobenzoate[3,5-bis(3,5-dimethylpyrazol-1-ylmethyl)toluene] of Co(II) ([Co(**L5**)(dnb)_2_]) (**6**)

A solution of **1** (0.42 mmol, 0.20 g) in ethyl acetate (10 mL) was added to a solution of **L5** (0.40 mmol; 0.12 g) in DCM (10 mL). The mixture was refluxed for 5 h. The solvent was evaporated to dryness to give a purple solid, which was washed with ethyl acetate: cyclohexene (20:30) and then with ethyl ether and dried at 100 °C for 8 h. Yield: 0.222 g (87%). IR (KBr, **υ**/cm^−1^): 3101, 2927, 1624, 1550, 1346, 1313, 925, 725. Raman (**υ**/cm^−1^): 1548, 1345, 1192, 1049, 996, 815, 579, 345, 207. Anal. calcd. for C_33_H_30_CoN_8_O_12_: C, 50.20; H, 3.83; N, 14.19%; Found: C, 49.96; H, 3.79; N, 14.16%. UV-Vis (DMSO) [(concentration, M) λ_máx_, nm (Log ε, M^−1^ cm^−1^)]: (3.37 × 10^−4^) 261 (3.46); (1.01 × 10^−3^) 538 (1.49).

#### 3.3.7. Synthesis of Dinitrobenzoate[3,5-bis(3,5-dimethyl-4-nitro-pyrazol-1-ylmethyl)toluene] of Co(II) ([Co(**L6**)(dnb)_2_]) (**7**)

A solution of **1** (0.50 mmol, 0.240 g) in ethyl acetate (10 mL) was added to a solution of **L6** (0.40 mmol, 0.16 g) in toluene (10 mL). The mixture was heated to reflux for 7 h. The solvent was evaporated to dryness to give a blue solid, which was washed with CHCl_3_: n-heptane (30:20) and then with ethyl ether and dried at 100 °C for 8 h. Yield: 0.290 g (84%). IR (KBr, **υ**/cm^−1^): 3093, 2924, 1627, 1539, 1408, 1465, 1338, 999, 717. Raman (**υ**/cm^−1^): 1534, 1416, 1345, 1190, 998, 815, 345. Anal. calcd. for C_33_H_28_CoN_10_O_16_: C, 45.06; H, 3.21; N, 15.92%; Found: C, 45.05; H, 3.20; N, 15.90%. UV-Vis (DMSO) [(concentration, M) λ_máx_, nm (Log ε, M^−1^ cm^−1^)]: (3.43 × 10^−4^) 269 (3.13); (1.03 × 10^−3^) 549 (2.03).

### 3.4. Biological Studies

#### 3.4.1. In Vitro Anti *T. cruzi* Activity

*T. cruzi* (SYLVIO-X10, ATCC (American Type Culture Collection)) were obtained from M. López-Casillas, Fundación Cardiovascular de Colombia. Epimastigotes were cultivated to the exponential growth phase in 96-well plates at a concentration of 5 × 10^5^ parasites/mL in LIT (liver infusion triptose) medium supplemented with 10% SFBi (serum bovine fetal inactivated) and incubated at 28 °C. Subsequently, the parasites were exposed to the different compound concentrations (100, 33.3, 11.1, 3.7 µg mL^−1^) for 72 h and evaluated in triplicate. Untreated parasites and parasites treated with benznidazole maintained under the same conditions were used as negative and positive controls, respectively. Benznidazole (purified by L.Y. Vargas, Universidad Santo Tomás, Santander, Colombia) was used as the reference drug. Growth inhibition was determined by optical microscopy using Trypan Blue (Sigma-Aldrich, Saint Louis, MO, USA). The antiparasitic activity of the compounds evaluated is expressed as the concentration required to inhibit 50% of parasites (IC_50_).

#### 3.4.2. In Vitro Antifungal Susceptibility Testing

The antifungal effect of the compounds against *Candida albicans* (ATCC^®^ 10231), *C. tropicalis* (ATCC^®^ 20366) and *C. krusei* (ATCC^®^ 14243) was determined. Strains were subcultured on Sabouraud dextrose and grown at 37 °C for 24 h prior to assays. Experiments were performed using the broth microdilution method according to the Clinical and Laboratory Standards Institute (CLSI) M27A-3 and M07-A10 protocols. Stock solutions (100 times higher than the highest tested concentration) were prepared using dimethyl sulfoxide (DMSO; Merck, Darmstadt, Alemania) to dissolve each compound. Intermedial dilutions were made in order to reduce the final solvent concentration to <1% using RPMI 1640 medium (Gibco, Life Technology, Carlsbad, CA, USA) supplemented with 3-(*N*-morpholino)propanesulfonic acid (MOPS, Sigma-Aldrich, Saint Louis, MO, USA). Twofold dilutions in the rank of 1.95–2000 µg mL^−1^ of each compound was tested in 96-well plates. Untreated controls were similarly evaluated. Itraconazole was purchased from Sigma-Aldrich (St. Louis, MI, USA) and used as the reference drug. Inoculum containing between 2500 and 5000 cells/mL of each strain of *Candida* was added to each well of the plate and incubated at 37 °C for 24 h. The MIC and fungicidal endpoint were determined as described previously (Murcia, R.A., et al., 2018). Triplicate measurements of each of the compound concentrations were obtained in two independent assays.

#### 3.4.3. Germ Tube Inhibition Assay

The effect of the complexes and their respective ligands on germ tube formation of *Candida albicans* was tested. The strain was subcultured on Sabouraud dextrose agar and grown at 37 °C for 24 h prior to assays. A loopful of inoculum was added into pooled human serum at a final concentration of 1 × 10^6^ yeast/mL. Further two-fold dilutions were prepared to inoculate each well of a 96-well plate containing 100 µL of pooled human serum with or without different concentrations of the compounds ranging from 500 to 62.5 µg mL^−1^. The test plates were incubated for 3 h at 37 °C. After the indicated time, the presence of a tube germ was investigated by counting at least 200 cells with a hemocytometer under the 40× objective lens. The positive control was established by supplementing human serum with Itraconazole (range of concentrations: 1.0–0.25 µg mL^−1^). The results are expressed as inhibition percentages of tube germ formation using the following formula: Control mycelium percent—mycelium percent of the treatment/Control mycelium percent × 100. For each concentration, five repetitions were performed.

#### 3.4.4. Cytotoxicity in Mammalian Cells

Epithelial cells derived from African green monkey kidney (Vero, ATCC, CCL-81) were obtained from M. López-Casillas, Fundación Cardiovascular de Colombia, and cultured in 96-well plates in Dulbecco’s modified Eagle medium (DMEM) (Life Technology, Carlsbad, CA, USA) supplemented with 10% Inactive Fetal Bovine Serum (SFBi) and incubated at 37 °C, 5% CO_2_ and 95% humidity for 24 h until monolayer formation. Subsequently, the cells were exposed to the different compounds for 72 h; four concentrations were evaluated in triplicate (300, 100, 33.3 and 11.1 µg mL^−1^). Cell viability was determined by using an MTT (3-(4,5-dimethylthiazol-2-yl)-2,5-diphenyltetrazole) colorimetric test as previously described (Leal-Pinto et al., 2019). The cytotoxicity percentage was calculated by the following equation: Cytotoxicity (%) = ((DO control group − DO treated group)/DO control group) × 100. The results are expressed as Cytotoxic Concentration 50 (CC_50_) and 90 (CC_90_), calculated by sigmoidal regression using the statistical software Msxlfit™, ID Business Solution, (Guildford, UK).

#### 3.4.5. Statistical Analysis

The values obtained in the germ tube inhibition assays were analyzed by oneway ANOVA followed by Tukey’s *post hoc* test for multiple comparisons. A significance level of 5% was adopted.

##### In Vitro anti-*T. cruzi* Activity

*T. cruzi* epimastigotes in the exponential growth phase from the SYLVIO-X10 (ATCC) strain were cultivated in 96-well plates at a concentration of 5 × 10^5^ epimastigotes/mL in LIT (liver infusion triptose) medium supplemented with 10% SFBi (serum bovine fetal inactivated) and incubated at 28 °C. Subsequently, the parasites were exposed for 72 h to the compounds at different concentrations (100, 33.3, 11.1, 3.7 µg mL^−1^), with triplicate evaluations performed. Untreated parasites and parasites treated with benznidazole maintained under the same conditions were used as negative and positive controls, respectively. Benznidazole was used as the reference drug. Growth inhibition was determined by optical microscopy using Trypan Blue (Gibco). The antiparasitic activity of the compounds evaluated is expressed as the concentration required to inhibit 50% of parasites (IC_50_).

##### Cytotoxicity in Mammalian Cells

Epithelial cells derived from African green monkey kidney (Vero, ATCC (American Type Culture Collection)) and CCL-81 (*Cercopithecus aethiops*) cells were cultured in 96-well plates in DMEM (Dulbecco’s modified Eagle’s medium) (Life Technology, CA, USA) supplemented with 10% of Inactive Bovine Fetal Serum (SFBi), 1000 µg mL^−1^ of penicillin and 100 µg mL^−1^ of streptomycin and incubated at 37 °C, with 5% CO_2_ and 95% humidity for 24 h until the monolayer formation. Subsequently, the cells were exposed to the different compounds for 72 h, and four concentrations were evaluated in triplicate (300, 100, 33.3 and 11.1 μg/mL). Cell viability was determined using an MTT colorimetric test (3-(4,5-dimethylthiazol-2-yl)-2,5-diphenyltetrazole)). The treated cells were reincubated for 4 h with MTT reagent (5 mg/mL), and, subsequently, the reduced formazan crystals were dissolved in DMSO. The optical density was determined via spectrophotometry at a wavelength of 595 nm, and the cytotoxicity percentage was calculated by the following equation: Cytotoxicity (%) = ((DO control group − DO treated group)/DO control group) × 100. From the inhibition percentages and respective concentrations, the results are expressed as Cytotoxic Concentration 50 (CC_50_), calculated by sigmoidal regression using the statistical software Msxlfit™, ID Business Solution (Guildford, UK).

## 4. Conclusions

In this paper, we report the synthesis and characterization of seven cobalt(II) complexes with ligands derived from pyrazoles and dinitrobenzoate. The ligand bis(3,5-dimethyl-4-nitro-1H-pyrazol-1-yl)methane (**L6**) and the metal complexes **1**–**7** are new. Ligand bonding to metal ions was confirmed by elemental analysis and Raman, infrared, ultraviolet/visible and mass spectrometry studies. The structures of **L5** and **1** were confirmed by X-ray diffraction analysis. The analyses’ spectral data and DFT calculations showed that complexes **2**–**7** had a 1:1:2 [*M(**L**)(dnb)_2_*] stoichiometry and octahedral geometries, while **1** had a 1:2 (*M:dnb*) stoichiometry. In general, the complexes showed higher antifungal activity than the free ligands under planktonic conditions. However, most ligands and **3** and Co(OAc)_2_ ∙4H_2_O complexes inhibited *C. albicans* filamentation in a dose-dependent manner. In addition, none of the tested compounds were toxic to Vero cells. These results indicate that the compounds are promising alternative inhibitors of important virulence factors during candidiasis. On the other hand, the complexes did not present trypanocidal activity. Studies to further elucidate their structure–activity relationship are in progress.

## Figures and Tables

**Table 1:** Analytical and physical data of the complexes.

Compound (Formula)	Molecular Weight/(g mol^−1^)	Color (Yield)/%	Melting Point/°C	Calculated (Found)/%		
				C	H	N
**1** (C_14_H_6_N_4_O_12_Co)	481.15	Purple (98)	300–302	34.95 (34.90)	1.26 (1.25)	11.46 (11.51)
**2** (C_25_H_22_N_8_O_12_Co)	685.43	Light pink (92)	324–326	43.81 (43.80)	3.24 (3.22)	16.35 (16.33)
**3** (C_25_H_20_N_10_O_16_Co)	775.42	Dark pink (90)	347–349	38.72 (38.64)	2.60 (2.60)	18.06 (17.99)
**4** (C_33_H_30_N_8_O_12_Co)	789.58	Purple (87)	353–355	47.95 (47.83)	3.50 (3.48)	16.23 (16.18)
**5** (C_33_H_28_N_10_O_16_Co)	879.57	Dark blue (84)	362–364	42.97 (42.69)	2.91 (2.90)	17.78 (17.73)
**6** (C_31_H_27_N_9_O_12_Co)	776.54	Pink (80)	378–380	50.20 (49.96)	3.83 (3.79)	14.19 (14.16)
**7** (C_31_H_25_N_11_O_16_Co)	866.54	Lilac (82)	384–386	45.06 (45.05)	3.21 (3.20)	15.92 (15.90)

**Table 2:** Infrared and Raman spectral bands of **1**–**7**. **υ**: stretching; **s**: symmetric; **as**: asymmetric; **δ**: flexion; **t**: tri-substituted.

Complex	FT-IR (cm^−1^)				Raman (cm^−1^)		
	υ_as_(NO_2_)	υ_s_(NO_2_)	υ_as_(COO^−^)	υ_s_(COO^−^)	δ_ring t_	υ(Co-O)	
**1**	1462	1346	1624	1577	999	346	204
**2**	1458	1342	1631	1589	996	339	201
**3**	1500	1342	1631	1570	998	341	208
**4**	1462	1346	1624	1581	996	345	207
**5**	1465	1338	1627	1604	996	343	206
**6**	1458	1338	1643	1573	993	348	212
**7**	1485	1342	1627	1566	996	348	207

**Table 3:** Crystal data and experimental details.

Complex	L5	1*m* (Methanol)	1*e* (Acetone: Ethanol)
***Crystal Data***			
Chemical formula	C_19_H_24_N_4_	C_18_H_22_CoN_4_O_16_	C_22_H_30_CoN_4_O_16_
*M*_r_	308.42	609.33	665.43
Crystal system, space group	Monoclinic, *C*2/*c*	Triclinic, *P*$\bar{1}$	Monoclinic, *P*2_1_/*n*
Temperature (K)	298	273	298
*a*, *b*, *c* (Å)	15.0005 (8), 8.4989 (6), 13.7852 (8)	6.4119 (13), 8.7752 (19), 12.154 (2)	6.7234 (4), 9.0108 (6), 24.6830 (13)
α, β, γ (°)	90.0, 96.778 (3), 90.0	90.444 (7), 100.387 (7), 102.212 (8)	90.0, 93.3622 (19), 90.0
*V* (Å^3^)	1745.16 (18)	656.7 (2)	1492.80 (15)
*Z*	4	1	2
Radiation type	Mo *K*α	Mo *K*α	Mo *K*α
µ (mm^−1^)	0.07	0.74	0.65
Crystal size (mm)	0.48 × 0.43 × 0.34	0.27 × 0.17 × 0.11	0.29 × 0.14 × 0.10
***Data Collection***			
Diffractometer	Bruker D8 Venture/Photon 100 CMOS diffractometer		
Absorption correction	Multi-scan SADABS; Bruker, 2016		
*T*_min_, *T*_max_	0.715, 0.747	0.700, 0.746	0.674, 0.746
No. of measured, independent and observed [I > 2σ(I)] reflections	39,902, 3342, 2036	24,349, 3987, 3301	32,804, 3050, 2439
*R*_int_	0.052	0.037	0.046
(sin θ/λ)_max_ (Å^−1^)	0.771	0.714	0.625
***Refinement***			
*R*[*F*^2^ > 2σ(*F*^2^)], *wR*(*F*^2^), *S*	0.057, 0.186, 1.03	0.042, 0.113, 1.03	0.045, 0.127, 1.09
No. of reflections	3342	3987	3050
No. of parameters	109	186	244
No. of restraints	0	24	80
H-atom treatment	H-atom parameters constrained	H atoms treated with a mixture of independent and constrained refinement	H atoms treated with a mixture of independent and constrained refinement
Δρ_max_, Δρ_min_ (e Å^−3^)	0.23 (0.91 Å from H2B), −0.24 (1.01 Å from N1)	0.53 (0.98 Å from O5), −0.39 (0.35 Å from O5)	0.33 (0.10 Å from C10A), −0.37 (0.19 Å from C11A)

**Table 4:** Antifungal and cytotoxic activity *.

Compound	*C. albicans*		*C. tropicalis*		*C. krusei*		Vero Cells	
	MIC	MFC	MIC	MFC	MIC	MFC	CC_50_ ± SD	CC_90_ ± SD
**L1**	1000	>2000	1000	>2000	1000	>2000	157.38 ± 3.1	>300
**L2**	>2000	>2000	>2000	>2000	>2000	>2000	>300	>300
**L3**	2000	>2000	2000	>2000	>2000	>2000	121.94 ± 2.1	>300
**L4**	>2000	>2000	>2000	>2000	>2000	>2000	111.43 ± 1.4	>300
**L5**	2000	>2000	2000	>2000	2000	>2000	44.95 ± 1.8	>300
**L6**	>2000	>2000	>2000	>2000	>2000	>2000	>300	>300
**1**	125	250	125	>500	62.5	>2000	197.05 ± 1.4	>300
**2**	125	250	125	>500	62.5	>1000	162.22 ± 2.5	>300
**3**	125	500	250	>2000	31.25	31.25	298.73 ± 3.6	>300
**4**	250	500	250	>2000	62.5	>2000	>300	>300
**5**	250	250	250	1000	62.5	>2000	>300	>300
**6**	250	>2000	125	>500	62.5	500	299.87 ± 3.2	>300
**7**	125	500	125	500	62.5	250	258.45 ± 1.4	>300
**Co(OAc)**_**2**_ **∙ 4H**_**2**_ **O**	125	125	125	250	31.25	>1000	103.97 ± 3.7	>300
**Itraconazole**	1.0	4.0	>16	ND	0.5	1.0	59.84 ± 4.5	>300

* MIC: Minimal inhibitory concentration; MFC: Minimum fungicidal concentration; CC_50_: Cytotoxic concentration 50. CC_90_: Cytotoxic concentration 90; SD = Standard deviation; ND: Not determined. Measurements are expressed in µg mL^−1^ and represent the average of two independent experiments with three repetitions.

**Table 5:** Inhibitory activity against *Candida albicans* germ tube formation.

Compound	Concentrations (µg mL^−1^) ± SD			
	500	250	125	62.5
**L1**	54.16 *^,†^ ± 6.82	16.35 ± 5.16	9.87 ± 1.48	NI
**L2**	43.47 * ± 7.18	39.55 * ± 2.86	7.82 ± 3.30	NI
**L3**	45.24 * ± 5.16	21.66 ± 3.83	NI	NI
**L4**	47.32 * ± 5.88	44.92 * ± 6.55	27.57 ** ± 4.43	NI
**L5**	33.28 * ± 7.03	30.65 ** ± 4.59	28.09 ** ± 5.99	25.21 ± 2.30
**4**	17.3 ± 1.64	NI	NI	NI
**5**	30.06 * ± 2.86	NI	NI	NI
**7**	12.01 ± 2.65	5.99 ± 2.21	4.56± 3.15	4.19 ± 3.18
Co(OAc)_2_ ∙ 4H_2_O	37.73 * ± 4.10	NI	NI	NI
**Concentrations (µg mL** ^**−1**^ **) ± SD**				
**Reference Compound**	1	0.5	0.25	0.125
**Itraconazole**	44.34 ± 6.15	39.91 ± 5.93	36.45 ± 2.80	ND

NI: Not inhibitory; ND: Not detected; SD: Standard deviation. The data represent the mean value of five replicates. * The effect of these compounds was equivalent to that of Itraconazole at 1 µg mL^−1^ (*p* > 0.05); ** the effect of these compounds on tube germ formation was statistically equivalent to that produced by Itraconazole at 0.5 µg mL^−1^ and 0.25 µg mL^−1^ (*p* > 0.05); ^†^ the effect of **L1** was better than that of the positive control at 0.5 and 0.25 µg mL^−1^ (*p* < 0.01).

## Supplementary Materials

The following are available online at https://www.mdpi.com/1422-0067/20/13/3237/s1, CCDC 1907575-1907577 contain supplementary crystallographic data for **L5**, **1*m*** and **1*e*,** respectively. These data can be obtained free of charge via http://www.ccdc.cam.ac.uk/conts/retrieving.html or the Cambridge Crystallographic Data Centre, 12 Union Road, Cambridge CB2 1EZ, UK; fax: (+44) 1223-336-033; or e-mail: deposit@ccdc.cam.ac.uk.
