# Supplementary material for: Inhibition of C. albicans Dimorphic Switch by Cobalt(II) Complexes with Ligands Derived from Pyrazoles and Dinitrobenzoate: Synthesis, Characterization and Biological Activity
Source: Int J Mol Sci. 2019 Jul 1;20(13):3237. doi: 10.3390/ijms20133237 (PMC6651002; doi:10.3390/ijms20133237)
Supplement: Supplementary file 1 [file ijms-20-03237-s001.pdf]

Supplementary Materials

Inhibition of *C. albicans* dimorphic switch by Cobalt(II) Complexes with Ligands Derived from Pyrazoles and Dinitrobenzoate: Synthesis, Characterization and Biological Activity

1. Characterization of ligands L1-L6

1.1 Bis(3,5-dimethyl-1-pyrazol-1-yl)methane (L1)

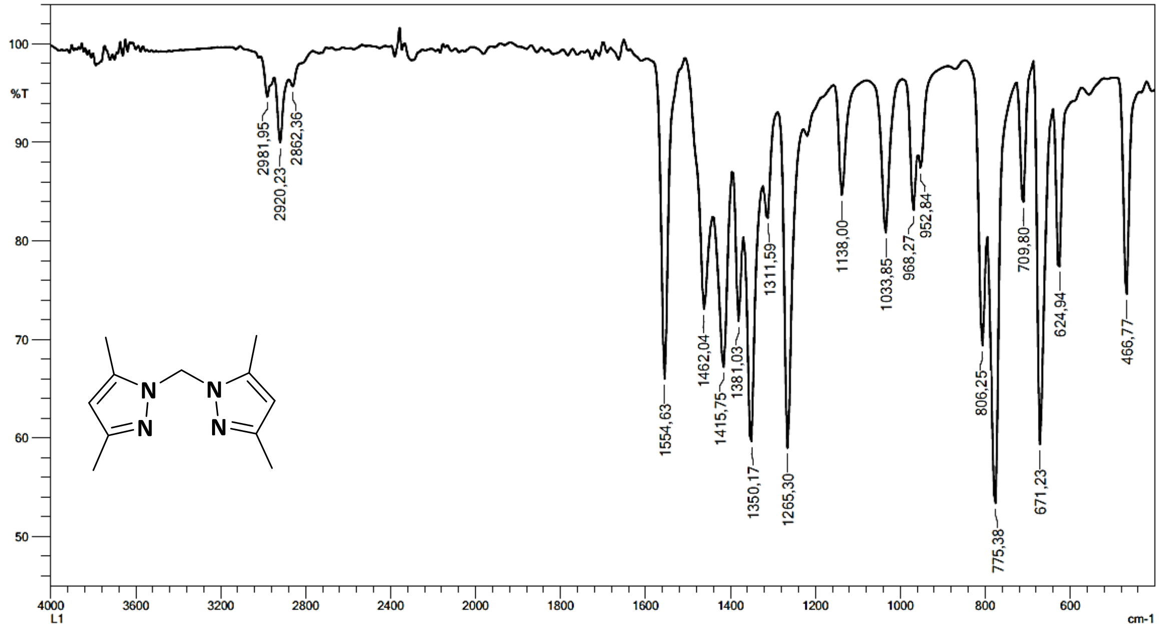

Figure S1. IR spectrum of **L1**.

1.2 Bis(3,5-dimethyl-4-nitro-1-pyrazol-1-yl)methane (L2)

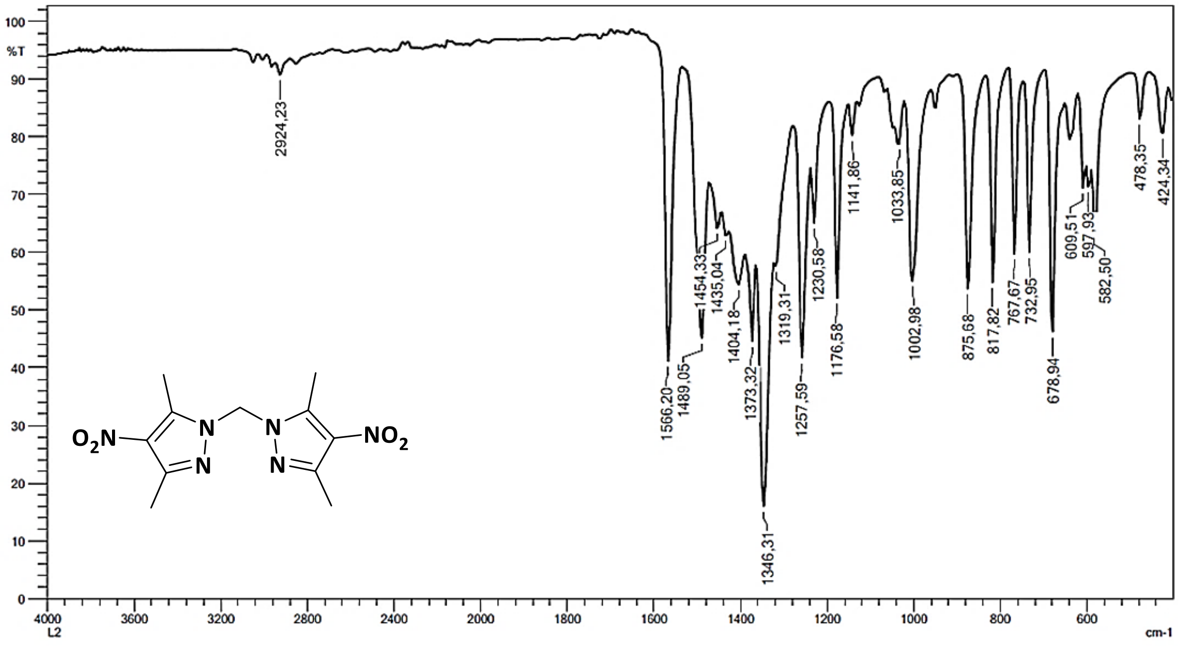

Figure S2. IR spectrum of **L2**.

1.3 2,6-bis(3,5-dimethylpyrazol-1-ylmethyl)pyridine (L3)

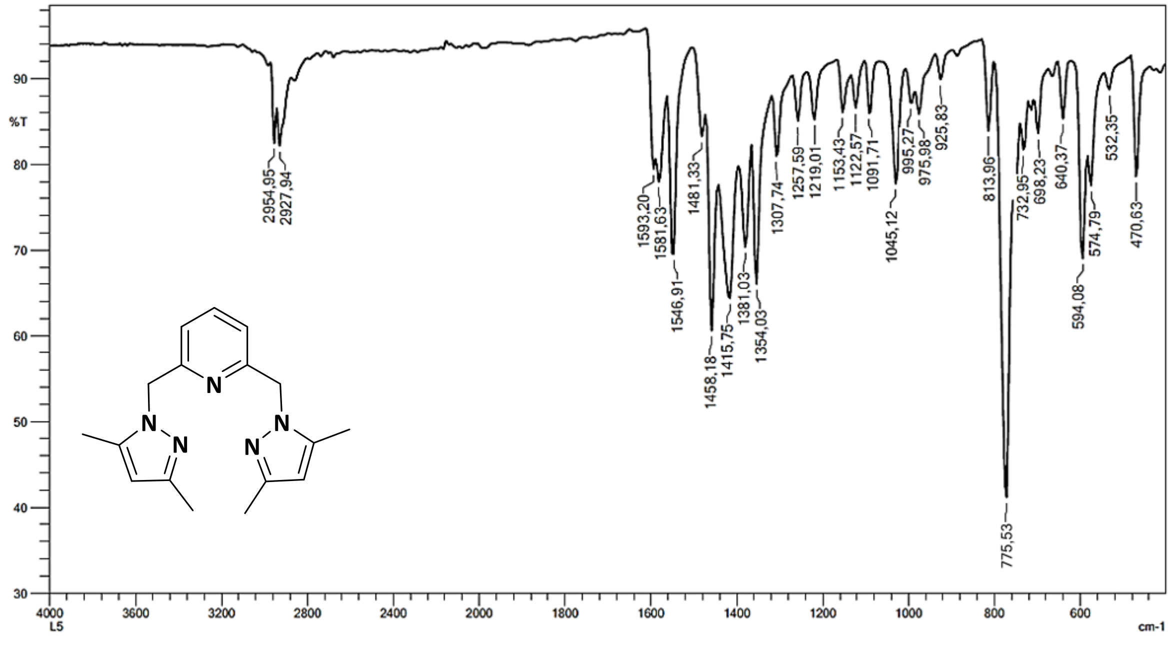

Figure S3. IR spectrum of **L3**.

1.4 2,6 bis (4-nitro-3,5-dimethylpyrazol-1-ylmethyl)pyridine (L4)

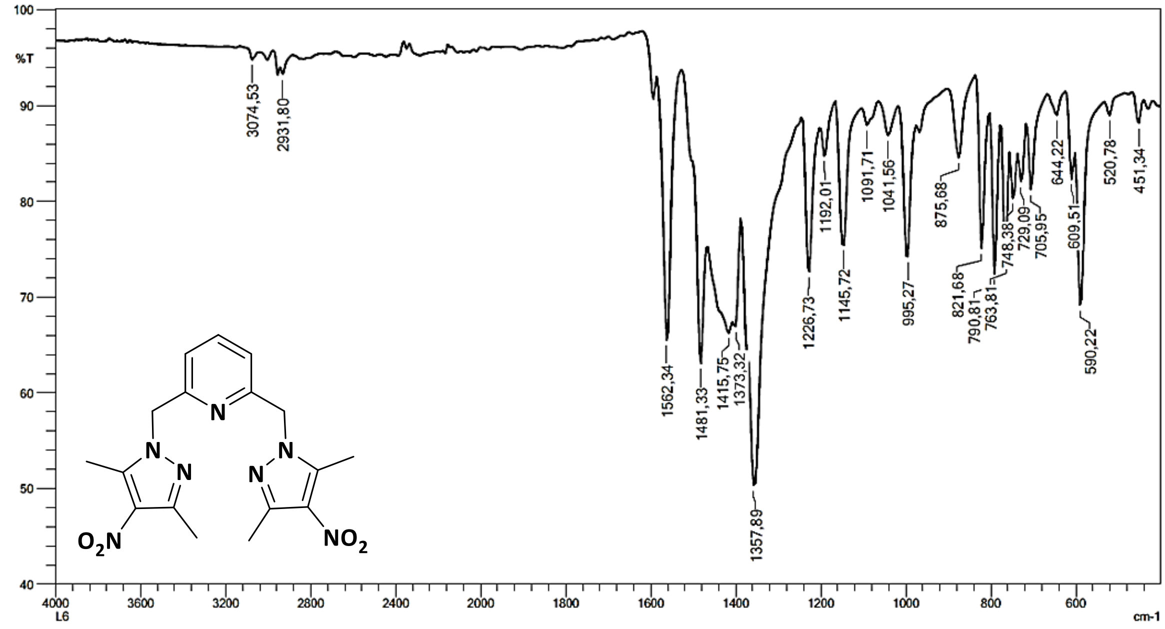

Figure S4. IR spectrum of **L4**.

1.5 3,5-bis(3,5-dimethylpyrazol-1-ylmethyl)toluene (L5)

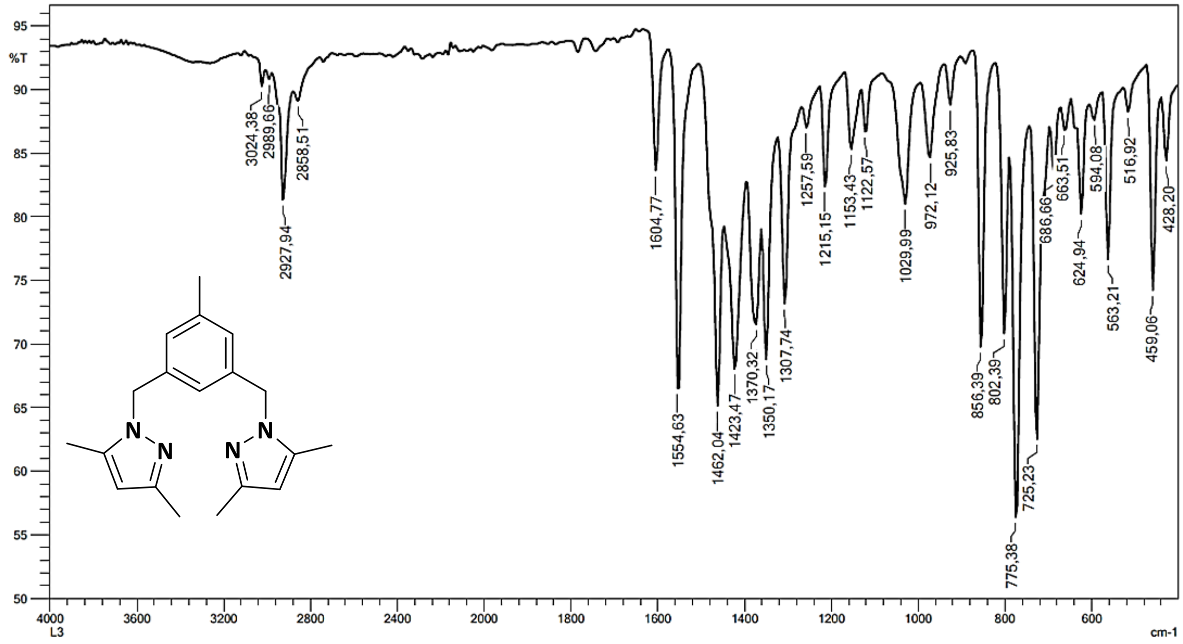

Figure S5. IR spectrum of **L5**.

1.6 3,5-bis(3,5-dimethyl-4-nitropyrazol-1-ylmethyl)toluene (L6)

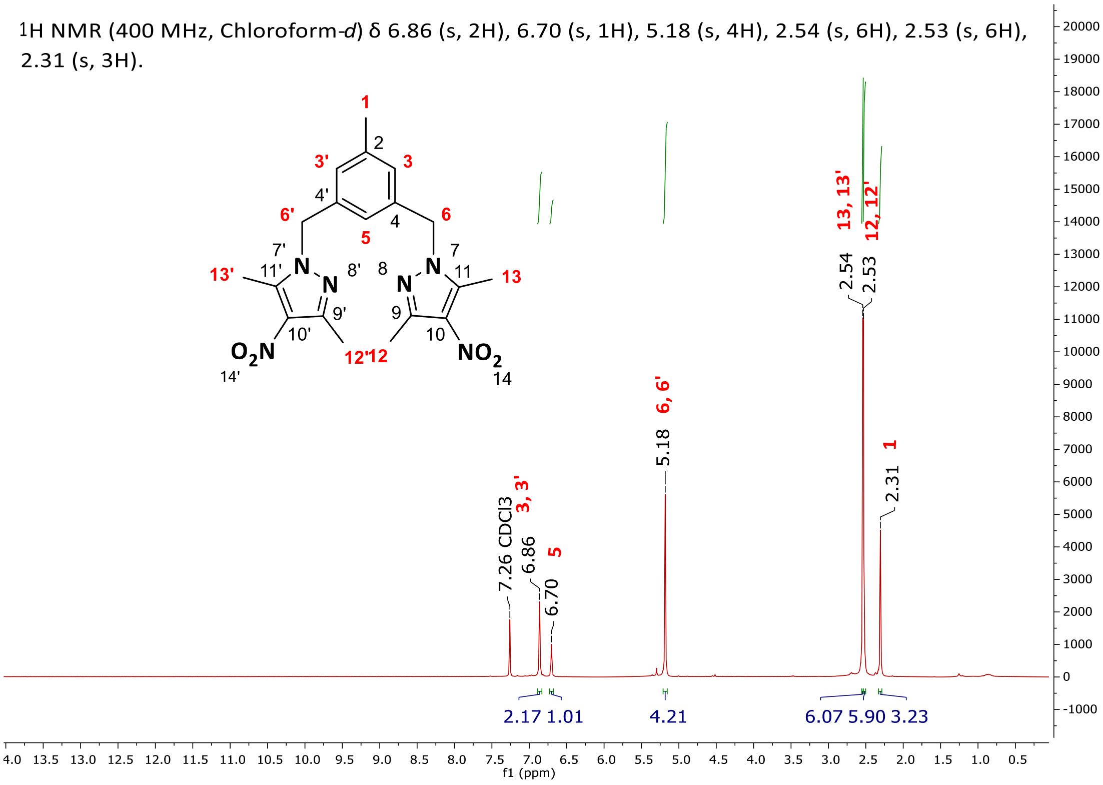

Figure S6. ^{1}H RMN spectrum of **L6.**

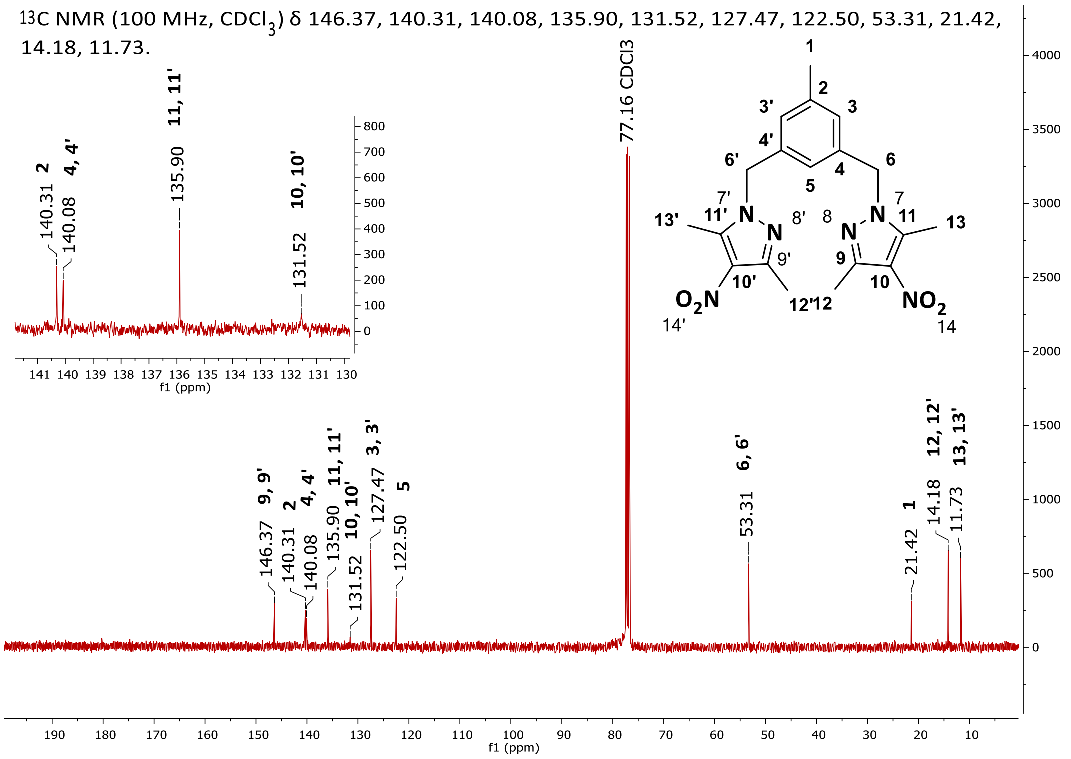

Figure S7. ^{13}C RMN spectrum of **L6**.

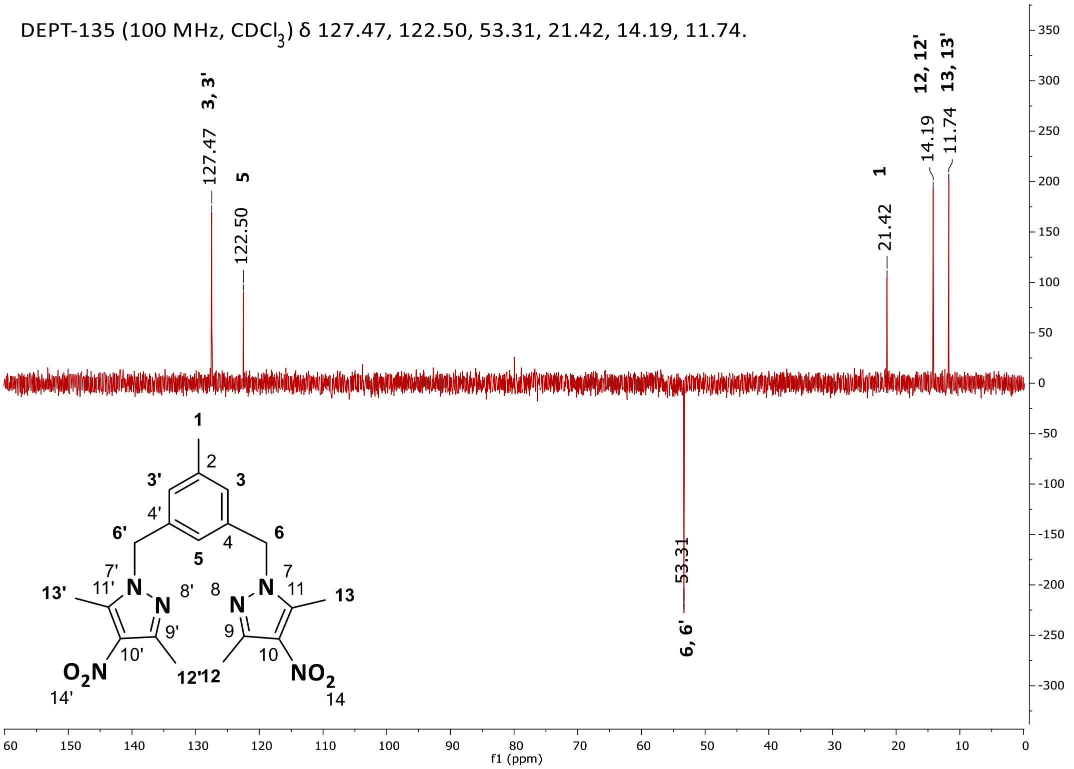

Figure S81. DEPT-135 spectrum of **L6**.

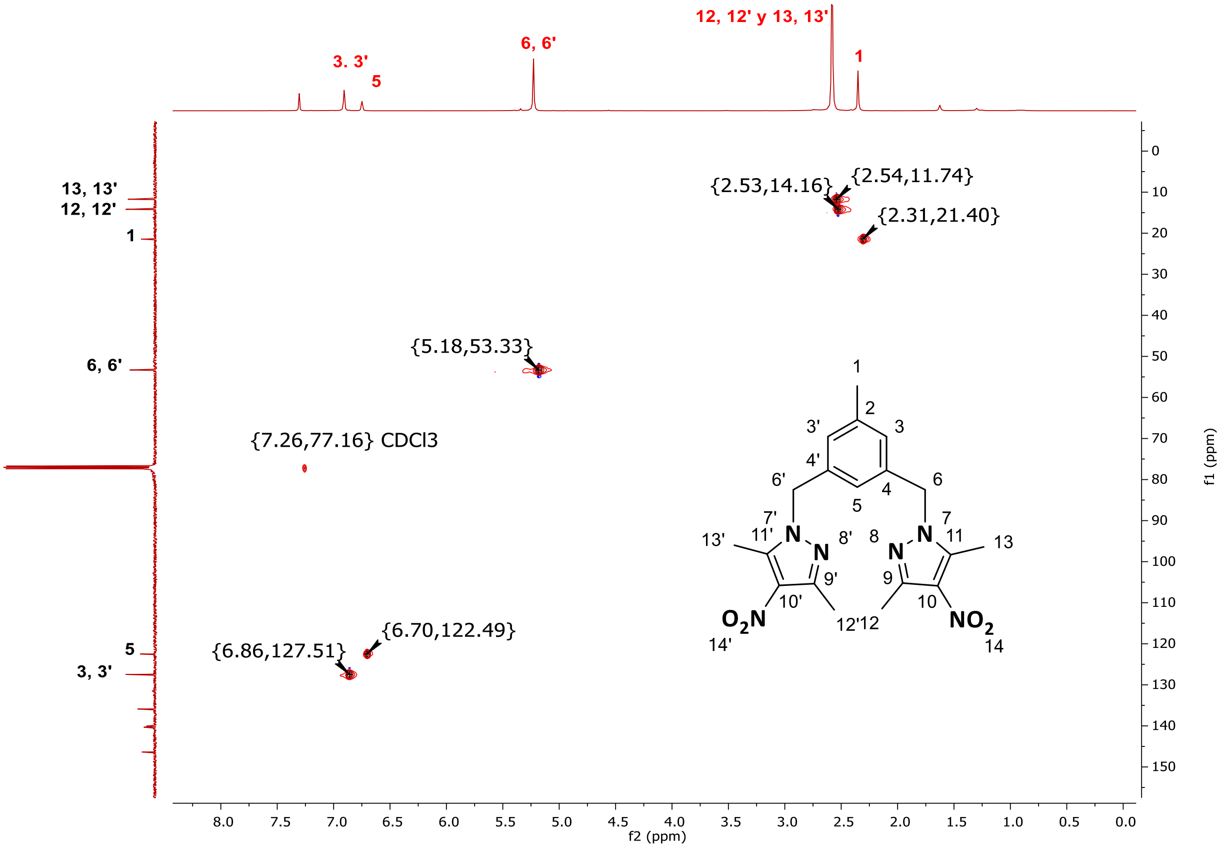

Figure S92. HSQC spectrum of **L6**.

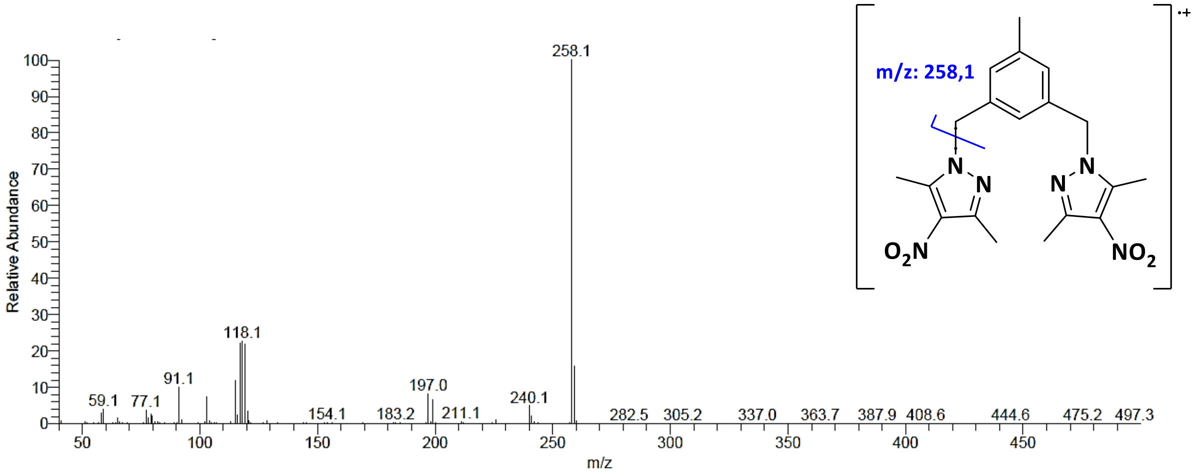

Figure S10. GC-MS spectrum of **L6.**

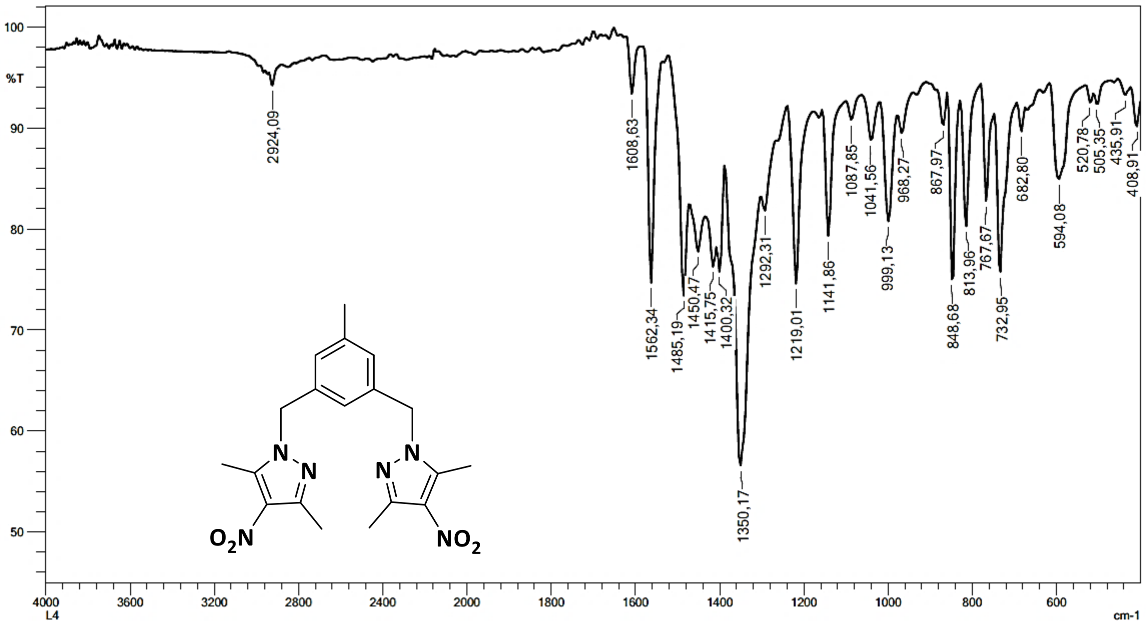

Figure S113. IR spectrum of **L6**.

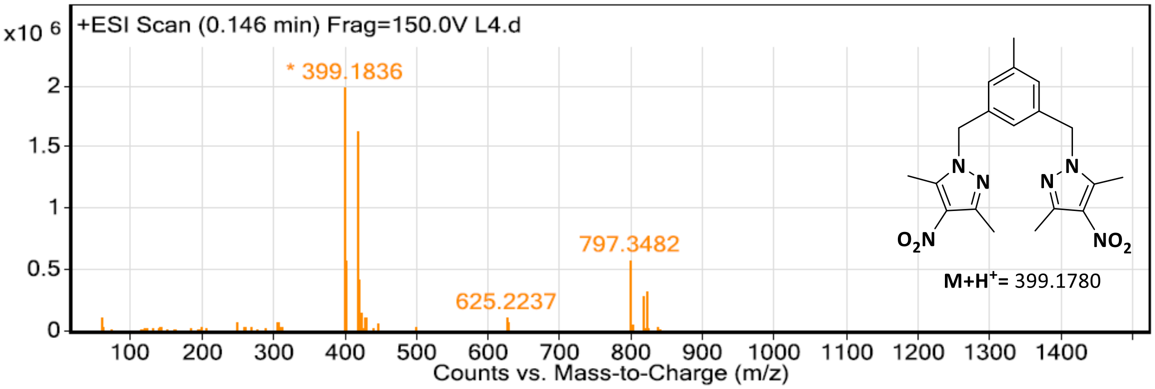

Figure S12. HRMS spectrum of **L6.**

2. Characterization of Complexes (1-7)

2.1 Bis(dinitrobenzoate-O, O ') of Co(II)

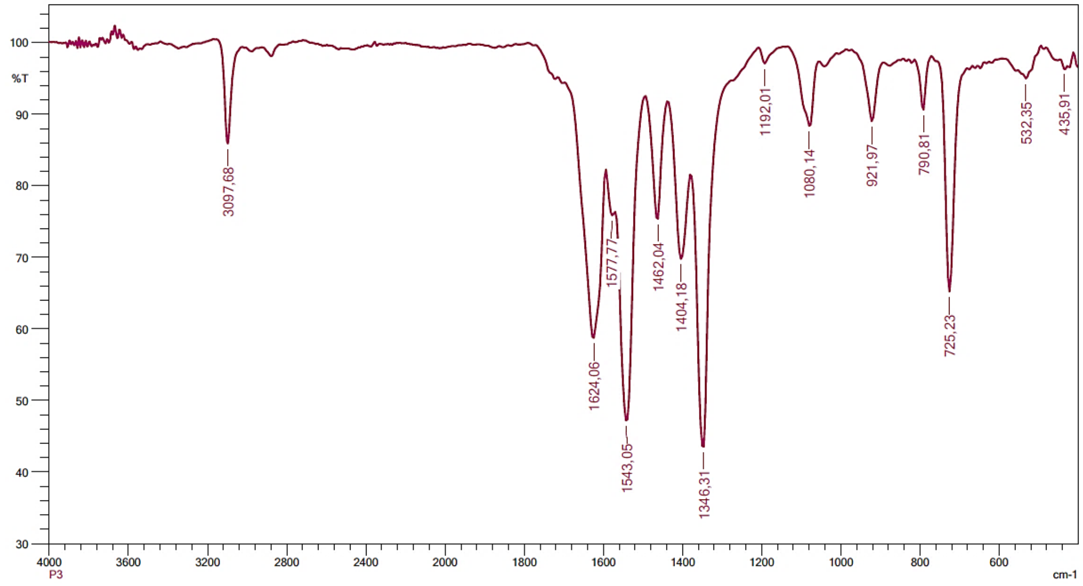

Figure S13. IR spectrum of **1**.

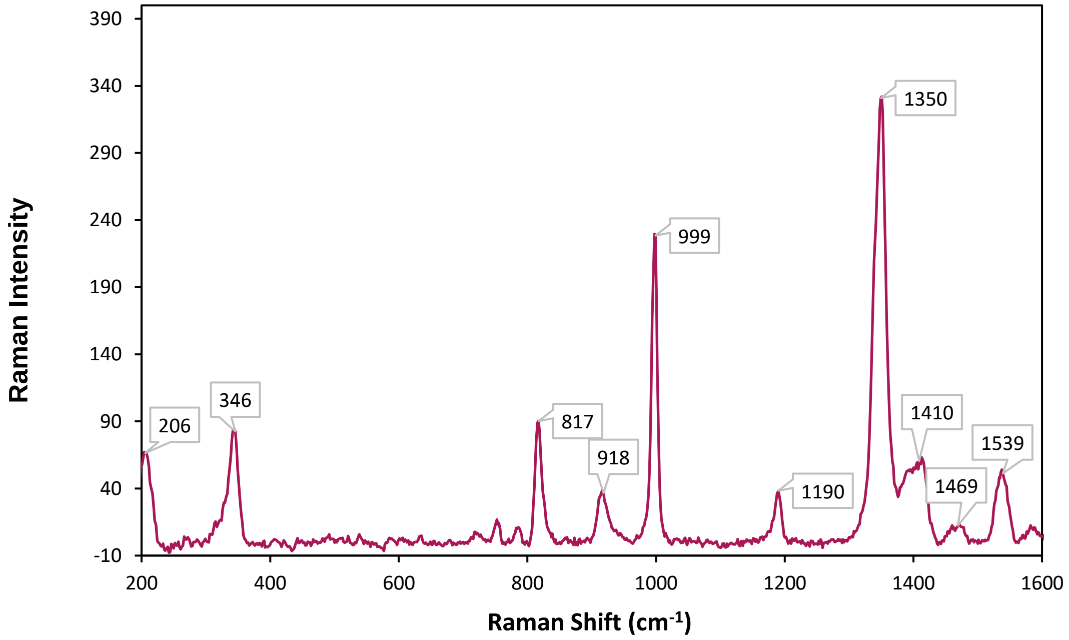

Figure S14. Raman spectrum of **1.**

2.2 Dinitrobenzoate[bis(3,5-dimethylpyrazol-1-yl)methane] of Co(II)

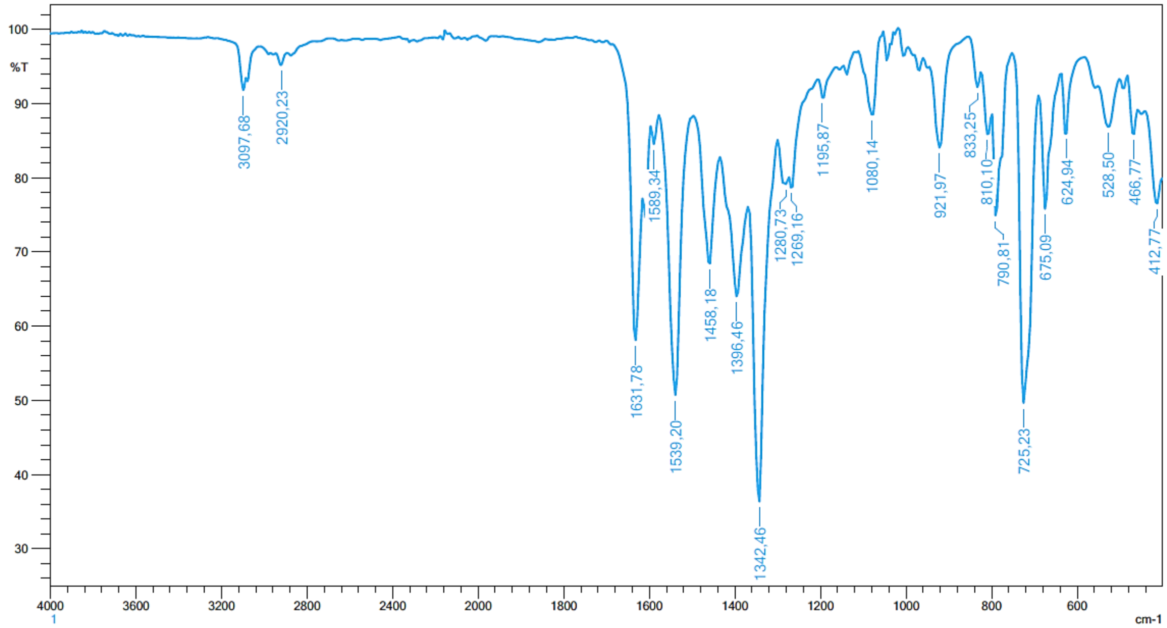

Figure S15. IR spectrum of **2**.

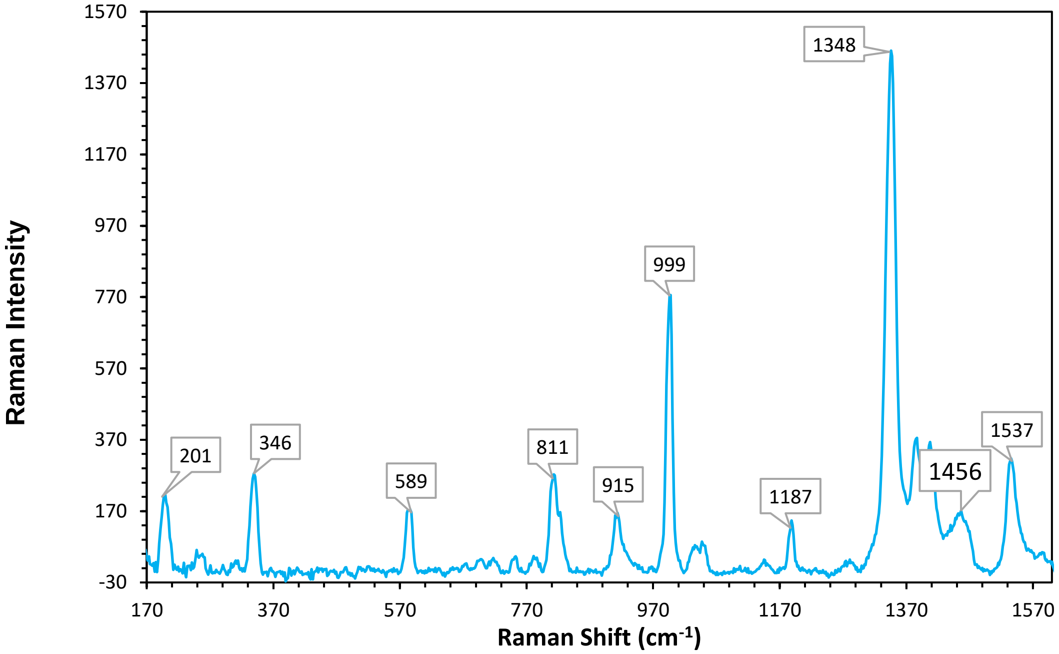

Figure S16. Raman spectrum of **2.**

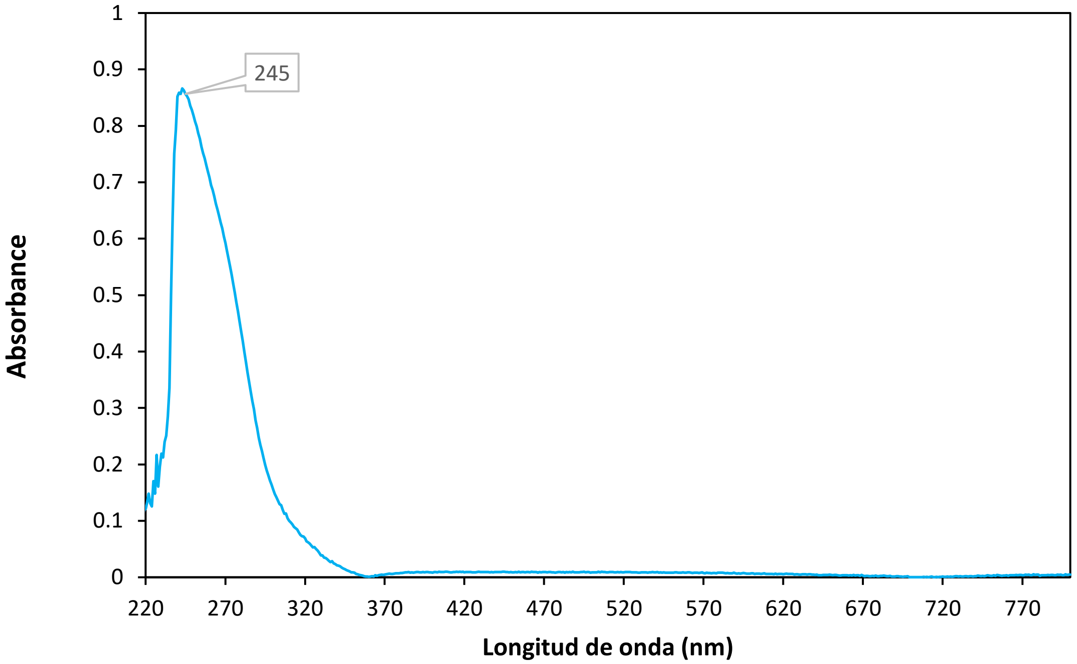

Figure S17. UV-Vis spectrum of 2.

2.3 Dinitrobenzoate[bis(3,5-dimethyl-4-nitro-1-pyrazol-1-yl)methane] of Co(II)

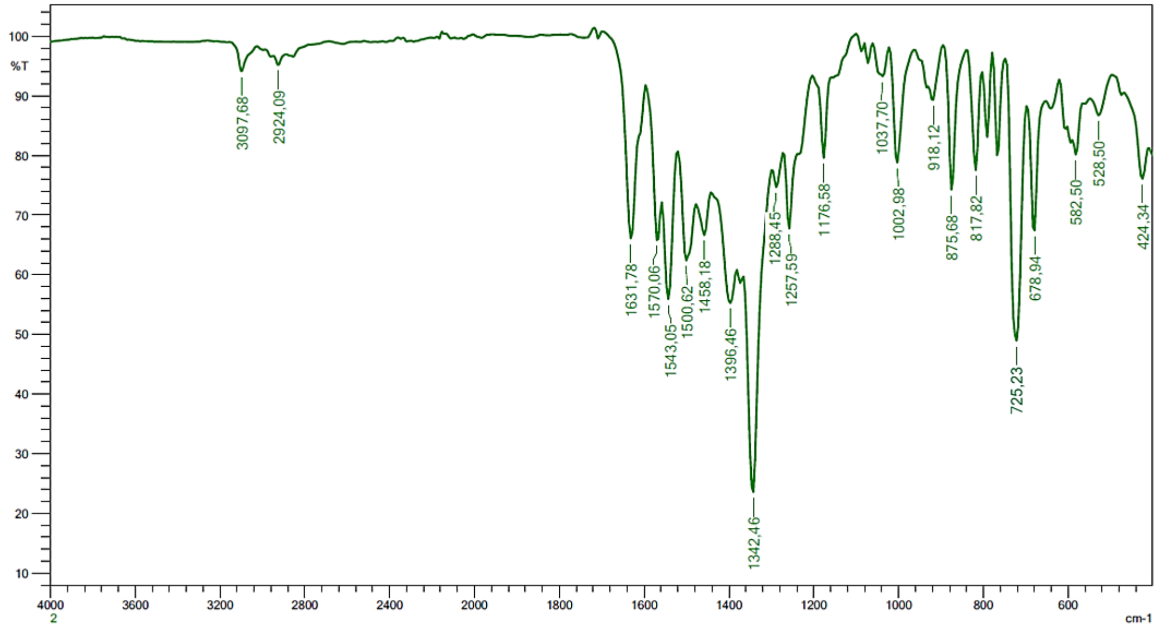

Figure S18. IR spectrum of **3.**

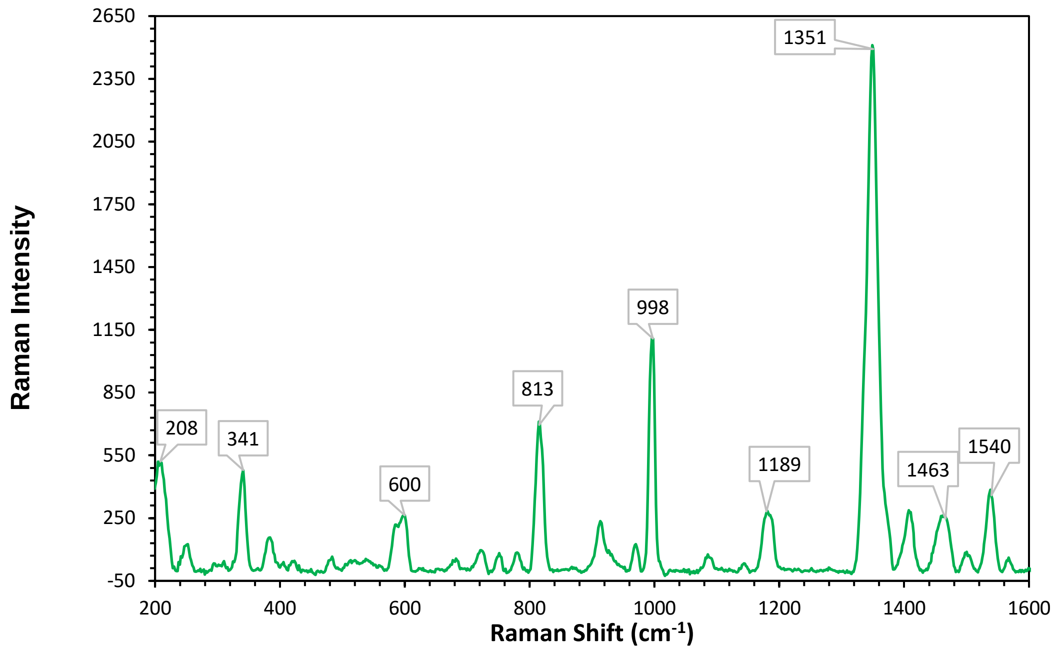

Figure S194. Raman spectrum of **3.**

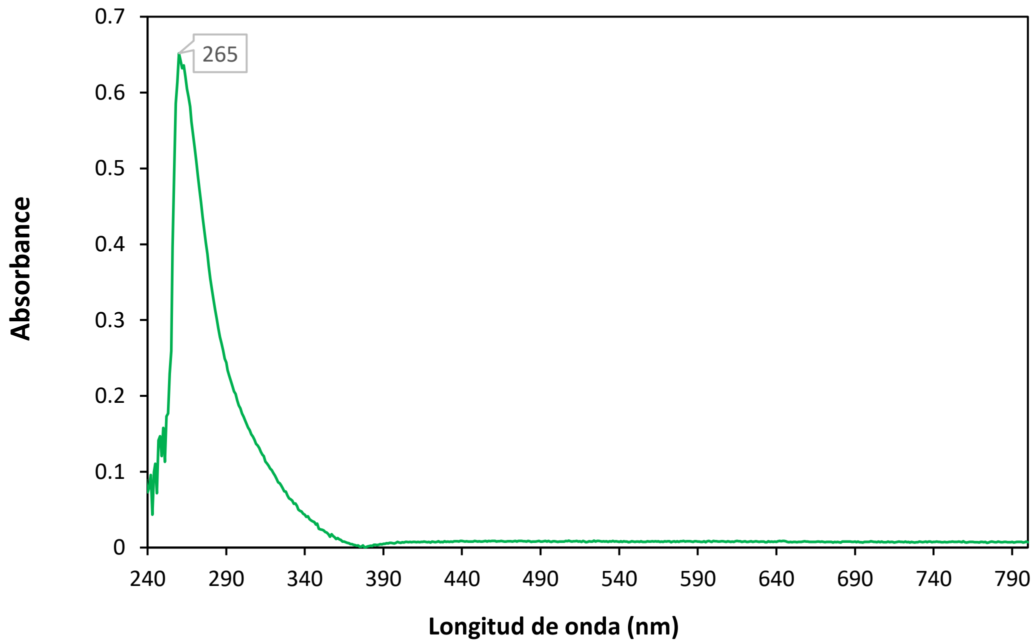

Figure S20. UV-Vis spectrum of 3.

2.4 [2,6-bis(3,5-dimethylpyrazol-1-ylmethyl)pyridine] of Co(II)

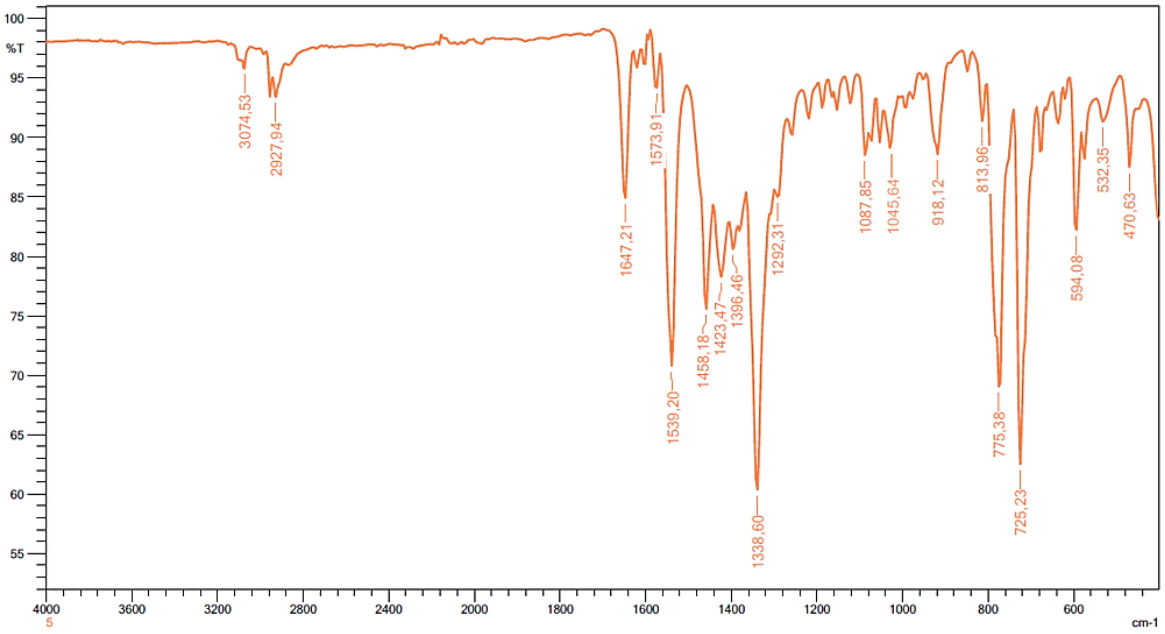

Figure S21. IR spectrum of **4.**

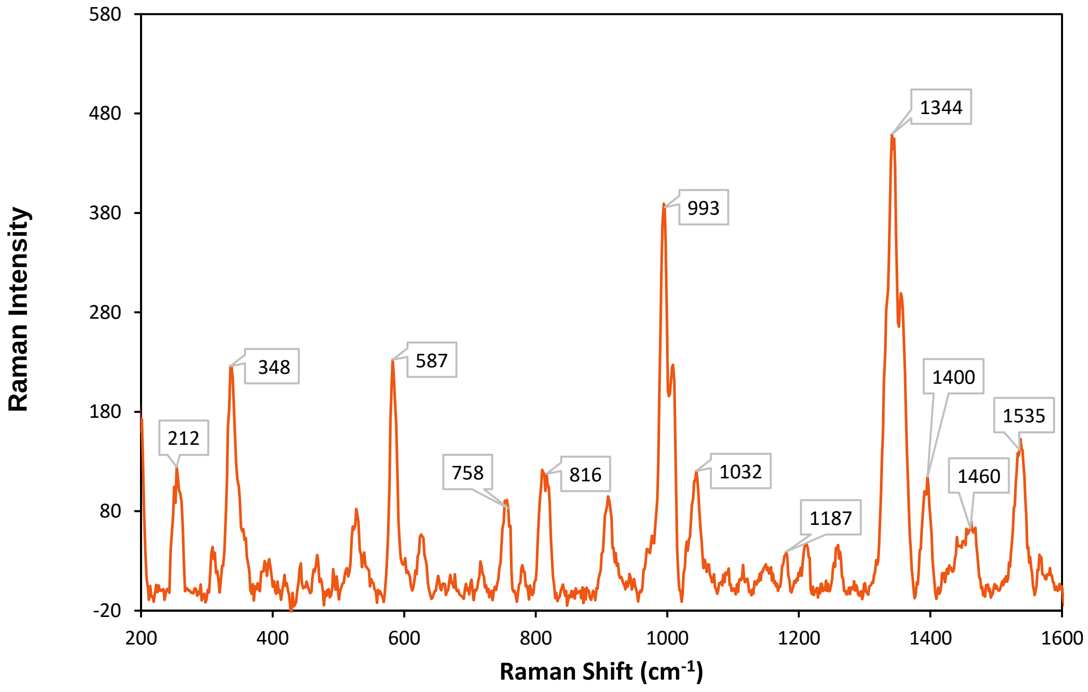

Figure S22. Raman spectrum of **4.**

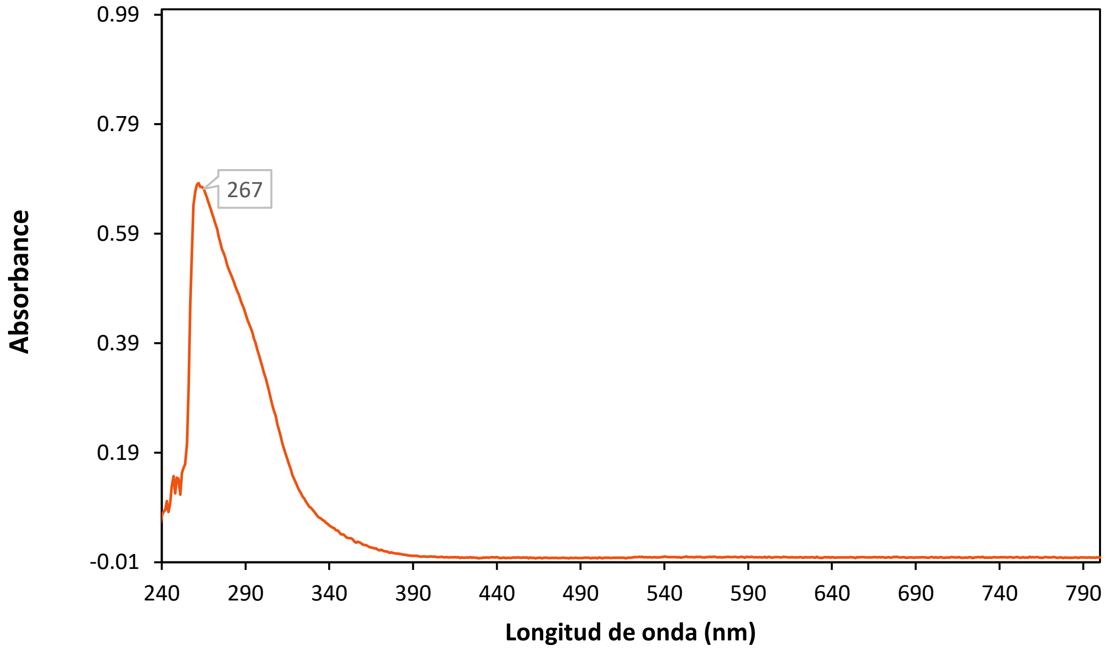

Figure S23. UV-Vis spectrum of **4.**

2.5 [2,6-bis(4-nitro-3,5-dimethylpyrazol-1-ylmethyl)pyridine] of Co(II)

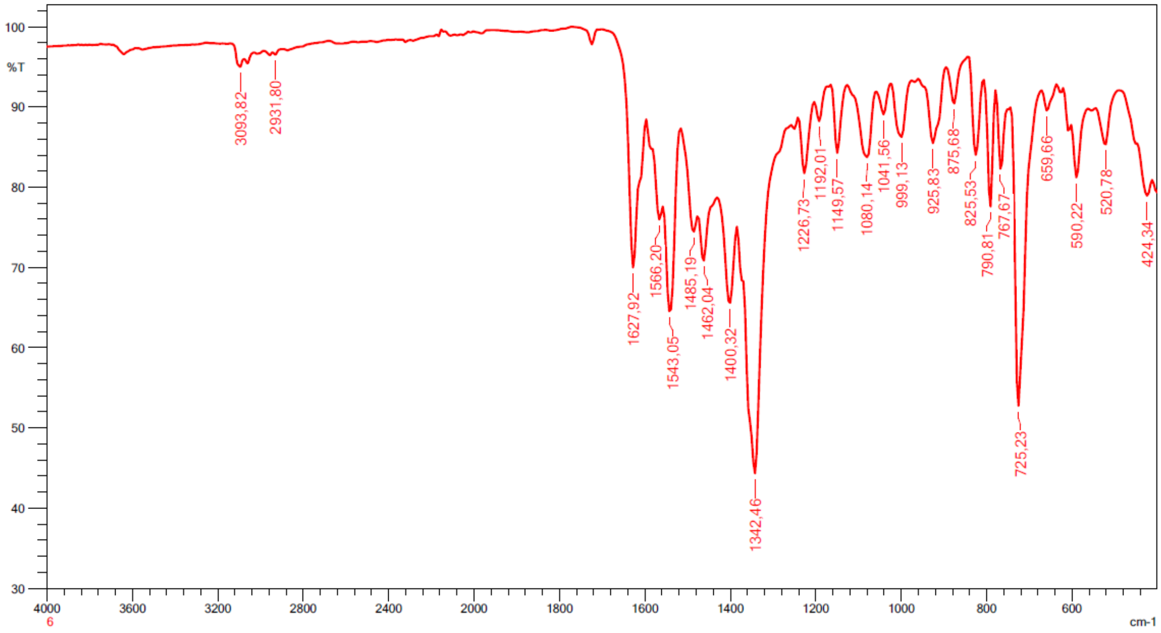

Figure S24. IR spectrum of **5.**

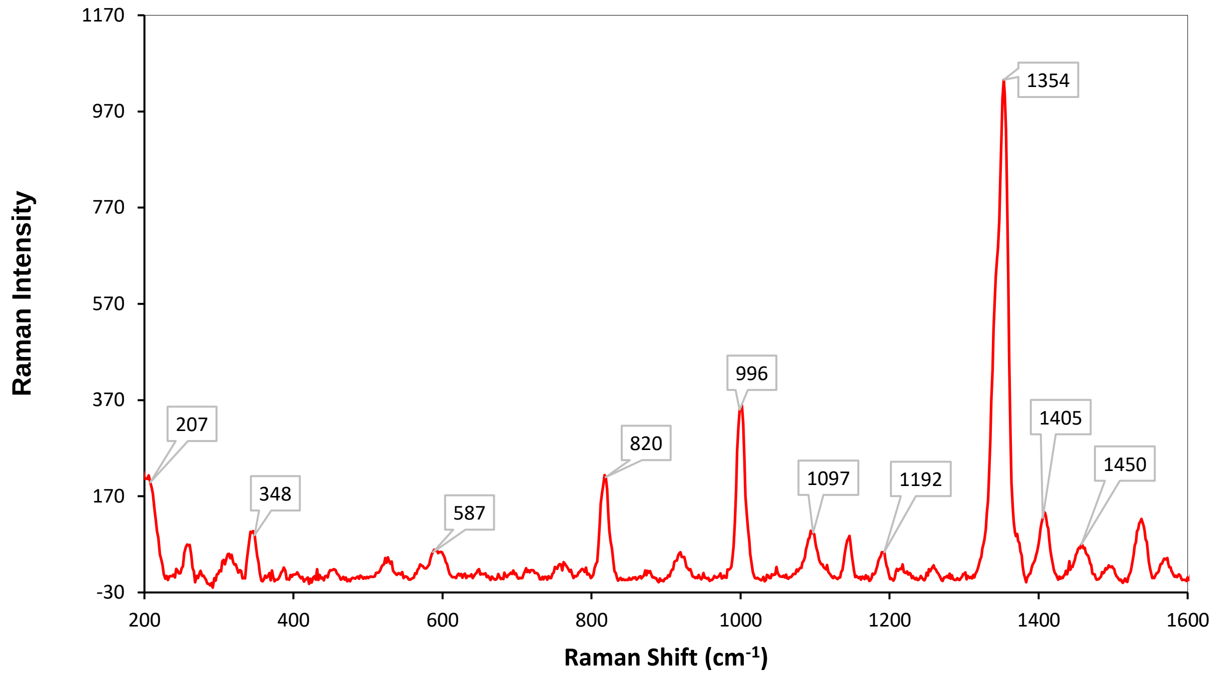

Figure S25. Raman spectrum of 5.

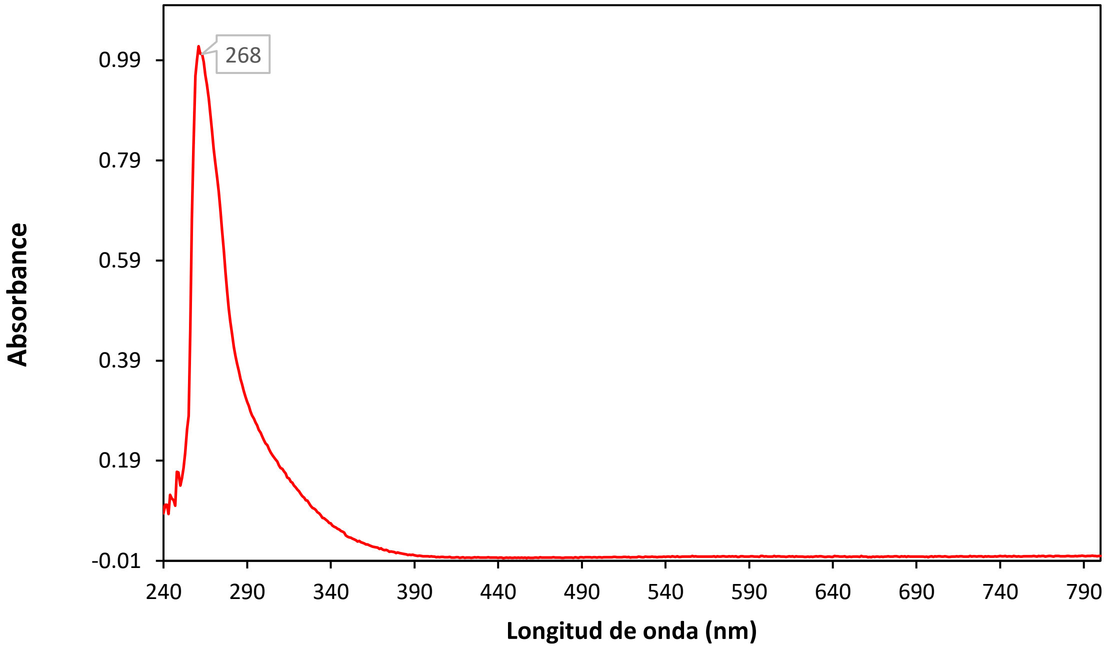

Figure S26. UV-Vis spectrum of **5.**

2.6 Dinitrobenzoate[3,5-bis(3,5-dimethylpyrazol-1-ylmethyl)toluene] of Co(II)

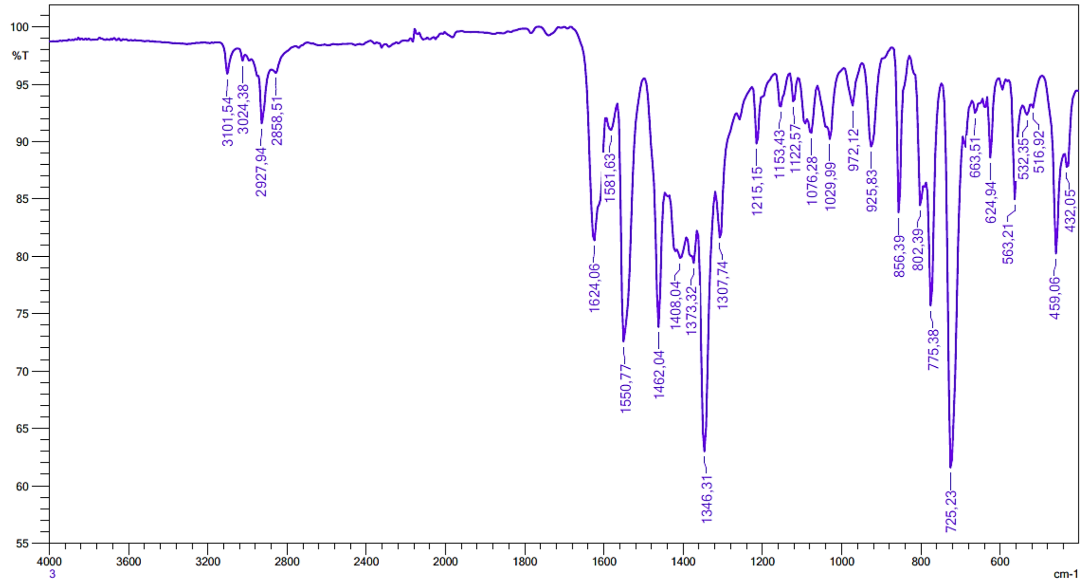

Figure S27. IR spectrum of **6.**

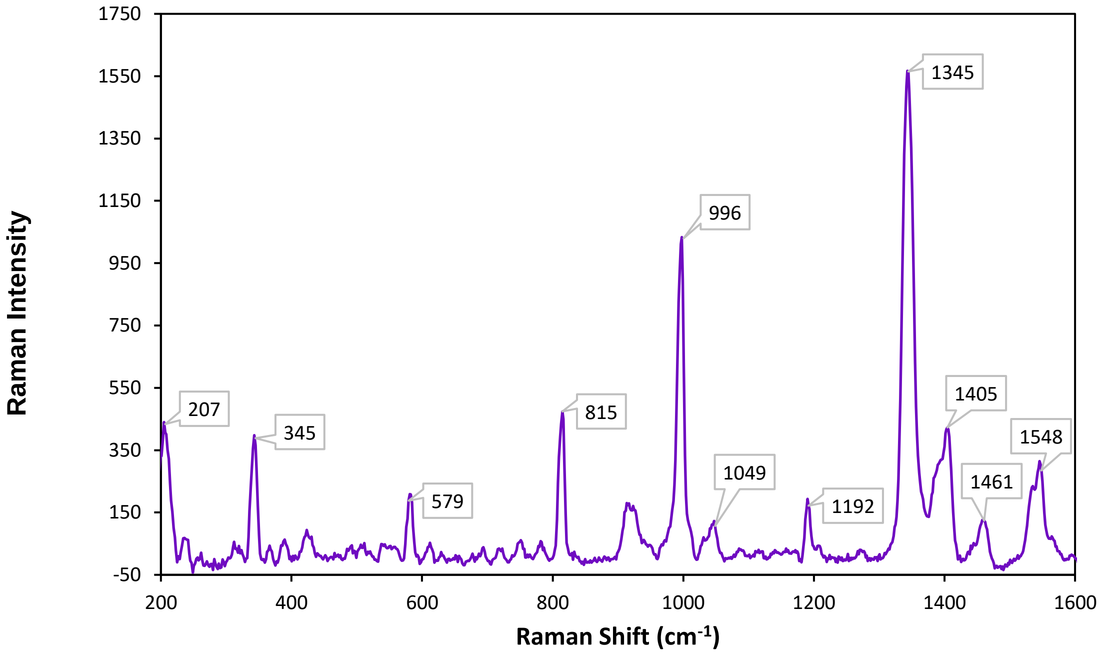

Figure S28. Raman spectrum of 6.

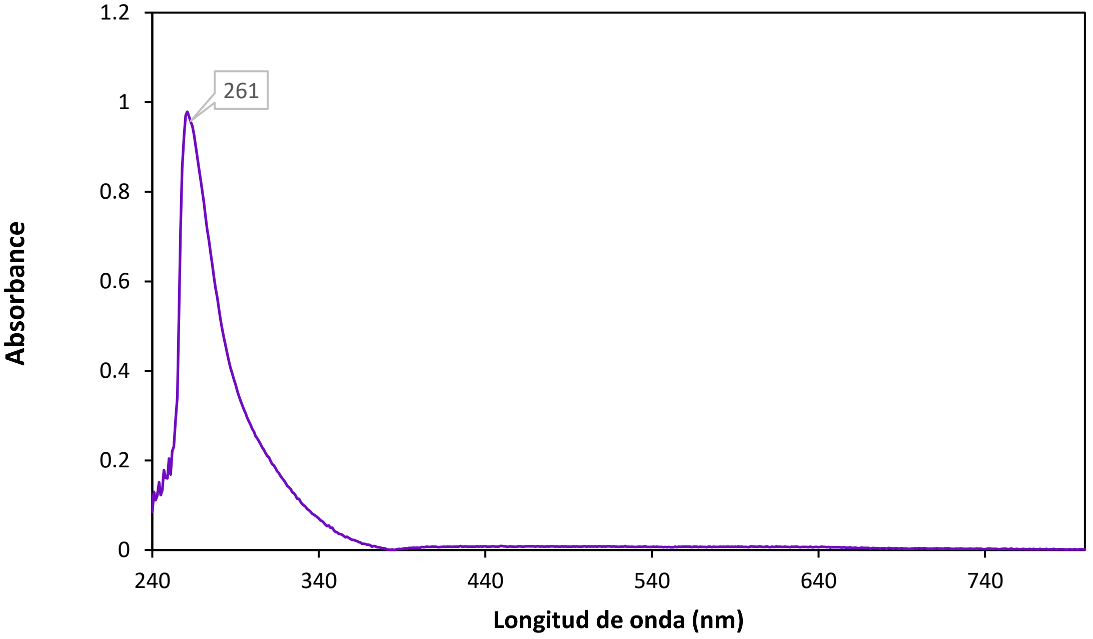

Figure S29. UV-Vis spectrum of 6.

2.7 Dinitrobenzoate[3,5-bis(3,5-dimethyl-4-nitropyrazol-1-ylmethyl) toluene] of Co(II)

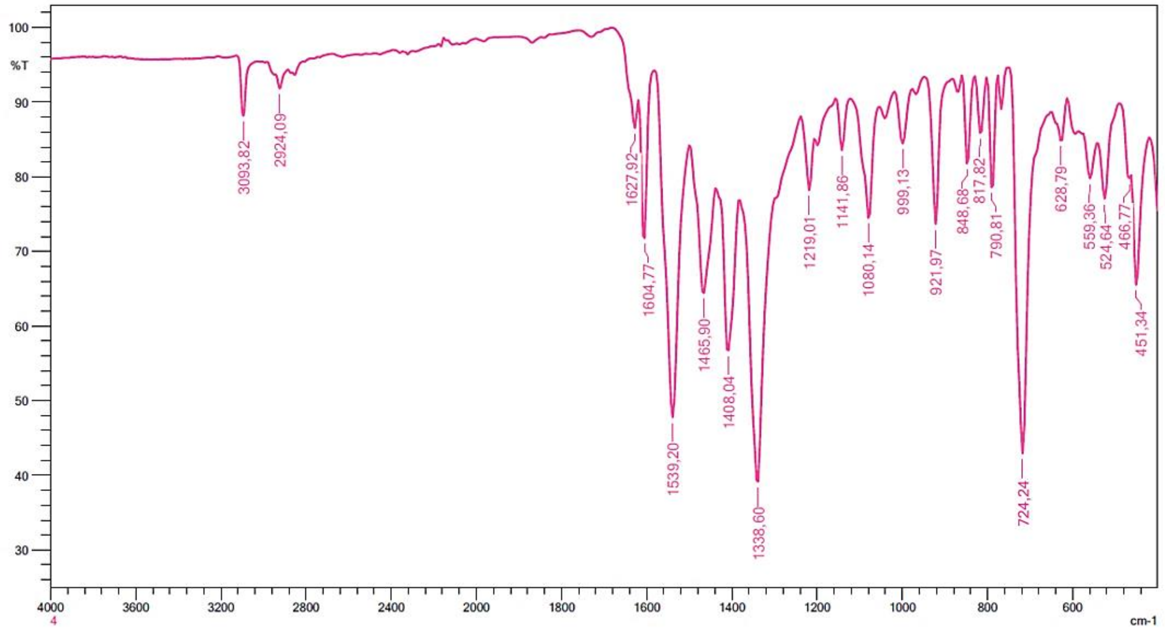

Figure S30. IR spectrum of **7.**

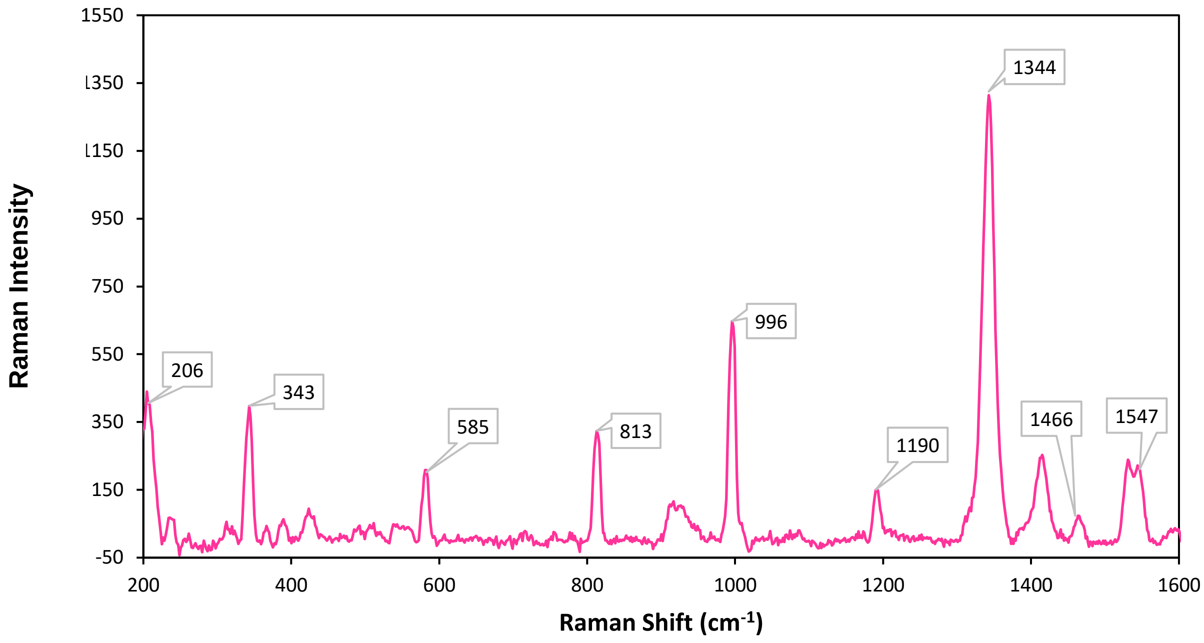

Figure S31. Raman spectrum of **7**.

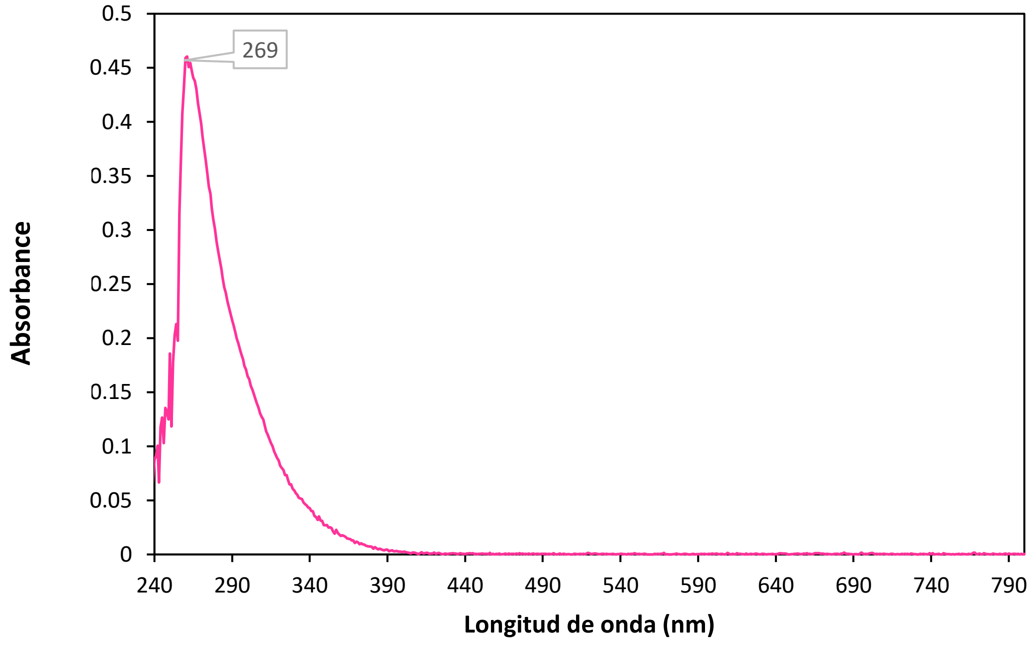

Figure S32. UV-Vis spectrum of **7**.

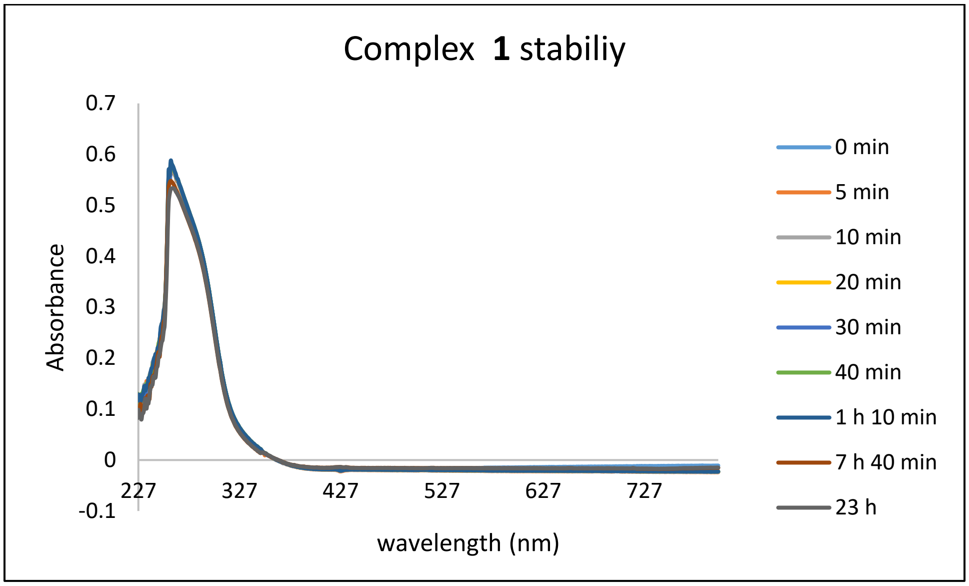

Figure S33. Stability study in solution for complex **1**. (In DMSO; 3 x10^{-5} M)

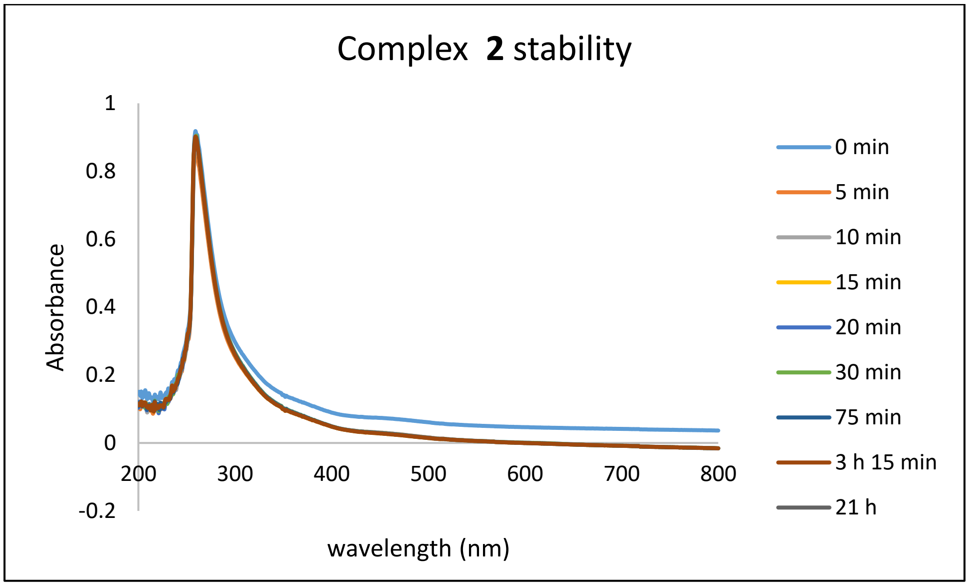

Figure S34. Stability study in solution for complex **2**. (In DMSO; 3 x10^{-5} M)

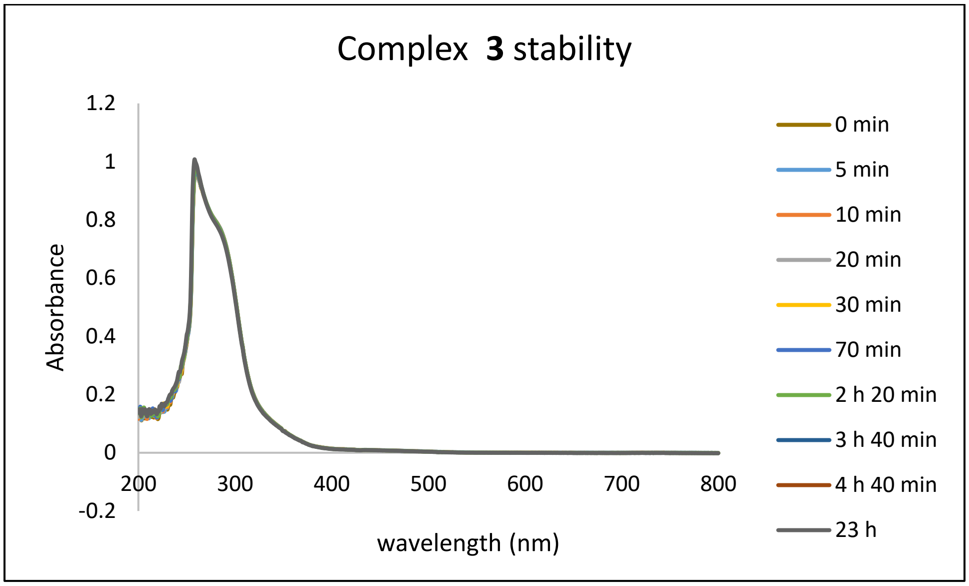

Figure S35. Stability study in solution for complex **3**. (In DMSO; 3 x10^{-5} M)

Table S1. Selected bands of the IR spectra (cm^{-1}) for **L1**-**L6** and **1**-**7**. υ: stretching; **as**: asymmetrical; **s**: symmetrical; **δ**: flexion; **op**: out of the plane; **Pz**: pyrazole.

Comp.	$υ_{CH_3}$ (C-H)	υ(C=C)	υ(C=N)	$δ_{Anillo\ Pz}$	$υ_{as}(NO_2)$	$υ_s(NO_2)$	$υ_{as}(COO^-)$	$υ_s(COO^-)$	$δ_{op}(C\text{-}NO_2)$
**7**	-	1543	-	-	1462	1346	1624	1577	725
**L1**	2920	1554	1381	806	-	-	-	-	-
**2**	2920	1539	1396	810	1458	1342	1631	1589	725
**L2**	2924	1566	1373	817	1489	1346	-	-	732
**3**	2924	1543	1396	817	1500	1342	1631	1570	725
**L3**	2927	1546	1381	813	-	-	-	-	-
**4**	2927	1539	1396	813	1458	1338	1647	1573	725
**L4**	2931	1562	1373	821	1481	1357	-	-	729
**5**	2931	1543	1400	825	1485	1342	1627	1566	725
**L5**	2927	1554	1370	802	-	-	-	-	-
**6**	2927	1550	1373	802	1462	1346	1624	1581	725
**L6**	2924	1562	1400	813	1485	1350	-	-	732
**7**	2924	1539	1408	817	1465	1338	1627	1604	725
